# Transitions from child to adult mental health care: the evidence-base for ESCAP guidance for clinicians

**DOI:** 10.1007/s00787-026-03036-0

**Published:** 2026-04-25

**Authors:** Helena Tuomainen, Rebecca Appleton, Gwendolyn C. Dieleman, Tomislav Franić, Giovanni de Girolamo, Athanasios Maras, Fiona McNicholas, Mathilde M. Overbeek, Diane Purper-Ouakil, Paramala Santosh, Ulrike M. E. Schulze, Cathy Street, Dieter Wolke, Swaran P. Singh

**Affiliations:** 1 Warwick Medical School, University of Warwick, Coventry, CV47AL UK; 2 School of Nursing and Midwifery, University of Worcester, Worcester, UK; 3 NIHR Policy Research Unit in Mental Health, Division of Psychiatry, University College London, London, UK; 4 Child and Adolescent Psychiatry/Psychology, Erasmus MC, Rotterdam, The Netherlands; 5 University of Split School of Medicine HR, University Hospital of Split, Split, Croatia; 6 IRCCS Centro San Giovanni di Dio Fatebenefratelli, Brescia, Italy; 7 ARQ National Psychotrauma Center, Diemen, The Netherlands; 8 School of Medicine & Medical Science, University College Dublin, Dublin, Ireland; 9 Lucena CAMHS, SJOG, Dublin, Ireland; 10 CHI Crumlin Dublin, Dublin, Ireland; 11 Clinical Child and Family Studies, Vrije Universiteit Amsterdam, Amsterdam, The Netherlands; 12 Yulius Mental Health Organization, Dordrecht, The Netherlands; 13 Child and Adolescent Psychiatry (MPEA1), CHU Montpellier, Montpellier, France; 14 INSERM, CESP, Villejuif, France; 15 Department of Child Psychiatry, King’s College London, London, UK; 16 Centre for Interventional Paediatric Psychopharmacology and Rare Diseases (CIPPRD), Maudsley Hospital, London, UK; 17 HealthTracker Ltd, Gillingham, Kent, UK; 18 Department of Child and Adolescent Psychiatry, Psychosomatics and Psychotherapy, University of Ulm, Ulm, Germany; 19 Department of Psychology, University of Warwick, Coventry, UK

**Keywords:** Systematic review, Youth mental health, Mental health services, Service transitions, Transitional care

## Abstract

The transition from Child and Adolescent Mental Health Services (CAMHS) to Adult Mental Health Services (AMHS) presents significant challenges, underscoring the need for improved transitional care procedures. Few European countries have implemented national transition-related guidance, despite the potential of clinical guidance to support appropriate care decisions and practices. We conducted a literature review to inform the development of the European Society of Child and Adolescent Psychiatry (ESCAP) transition guidance for clinicians. Following systematic principles, four databases (Medline, Embase, PsychInfo, Web of Science) were searched to identify relevant international research published from January 1, 2019 to April 10, 2025, to build on existing evidence. Titles and abstracts were reviewed by two independent reviewers. We screened 12,595 records and included 149 reports published since 2019. Illness severity was the primary predictor of AMHS transition, with only 20–25% transitioning directly, challenging assumptions that only specific diagnostic groups warrant continuity. Among those reaching the upper age boundary, the majority (40–60%) discontinue specialist services rather than transitioning to AMHS. Longitudinal data from the MILESTONE study – though limited by recruitment bias towards less severe cases – show that most maintain stable mental health without ongoing specialist input, yet 25% experience deterioration and 13% require readmission within two years. Many studies reported shortcomings regarding transition experiences for young people and parents/caregivers. However, structured preparation and stakeholder involvement can improve outcomes regardless of service destination. Findings challenge deficit-focused perspectives by showing that discontinuation represents the appropriate pathway for about half of young people. Substantial cross-country variation, often resource driven, underscores the importance of individualised, developmentally appropriate approaches that respect young people’s autonomy while providing necessary support.

**Supplementary Information:**

The online version contains supplementary material available at https://doi.org/10.1007/s00787-026-03036-0.

## Introduction

Child and adolescent mental health services (CAMHS) and adult mental health services (AMHS) in Europe typically operate as separate entities with distinct staff, procedures and service cultures [1]. Historical, societal and health systems developments have contributed to profound clinical, conceptual and ideological differences between these services [1–4]: CAMHS address not only neurodevelopmental and severe mental disorders in children, but also a broader spectrum of emotional and behavioural problems, including those related to parenting difficulties such as abuse and neglect. CAMHS usually work with families and collaborate closely with schools and other universal services, with entry criteria that are less restrictive but shaped by the need for parental consent for those under 18. In contrast, AMHS traditionally focus on severely ill patients with diagnosed mental disorders and operate with more restrictive entry criteria, reflecting limited resources, funding constraints, and high caseload pressures rather than clinical preference. This reduced capacity, combined with less experience managing early-onset conditions, can contribute to poor transition experiences [2, 5, 6].

The legal transition age varies between European countries (16–20 years), with most CAMHS setting an upper age limit of 18 years [3, 7]. However, service boundaries often create care gaps - some CAMHS terminate before young people can access AMHS, while discharge timing frequently reflects service thresholds rather than clinical readiness [8]. Flexible CAMHS age boundaries may mask young people remaining in services due to unavailable adult services rather than planned, age-appropriate care [9, 10].

The transition timing is particularly problematic given that late adolescence represents a period of heightened vulnerability. Young people face increased risks of cognitive, affective, and addictive disorders [11–15], while simultaneously navigating identity formation, educational transitions, and increasing independence [16–18]. Turning 18 means changes in legal power and authority and new autonomy, which is why caregiver involvement is reduced after the boundary [19]. This aspect is also something many young people and their parents/caregivers are not prepared for [20, 21]. Poor transitions with care interruptions can disrupt educational and vocational pathways, strain relationships, and negatively impact mental health outcomes [20, 22, 23].

Despite widespread recognition of care continuity for young people as top priority [24], European expert surveys reveal significant disconnection between CAMHS and AMHS [3, 7]. The child-adult mental healthcare divide extends to psychiatry training in Europe [25] where transition receives minimal attention [25–27], creating knowledge gaps that contribute to transition barriers [28]. While over two thirds of European countries have official national child and adolescent mental health policies, only four have policies or guidelines managing the CAMHS-AMHS interface (Cyprus, Denmark, Spain and the United Kingdom [UK]) [3, 7]. The UK’s National Institute for Health and Care Excellence (NICE) has published transition guidelines between paediatric and adult health care settings [29], though these are not specific to mental health. Without dedicated transition guidance, most clinicians rely on individual judgement, leading to inconsistency in care.

The ESCAP guidance on transitional care, published in this journal issue [30], addresses this gap by providing practical guidance for professionals involved in mental health service transitions across Europe. This paper presents the comprehensive systematic literature review that has informed the guidance development.

## Methods

Clinical guidance development combines literature evidence and expert opinion [29, 31]. The ESCAP guidance is based on European and international research on mental health service transitions from three sources: (1) recent systematic reviews associated with transitional care experiences and outcomes [5, 19, 32–35], (2) clinical transition-related guidelines (e.g. [29]).; and (3) systematic database searches.

The following questions guided the literature search and review process:

- What is the likelihood and what are the predictors of transition from child/adolescent to adult mental health services?
- What are the service use destinations for young people who reach the upper age boundary of their CAMHS?
- What are the mental health outcomes of young people transitioning to AMHS, staying in CAMHS and of those whose care is discontinued?
- What are the experiences of young people, parents/caregivers and health care professionals regarding transition?
- What are facilitators of positive transition experiences?
- What actions and interventions can improve transitional care, experiences and outcomes implemented within existing service structures?

Building upon evidence from our previous transition-related systematic reviews [5, 32] and studies [5, 21, 28, 36–43], we developed a comprehensive search strategy based on approaches by Appleton et al. [32] and Anderson et al. [33]. Four databases were searched: Medline, PsychINFO, Embase, and Web of Science. The initial search was conducted on 30 August 2023, with an updated search performed on 10 April 2025.

Database searches covered literature published from 1 January 2019 onwards, though notable earlier studies are also cited where relevant to provide historical context. Additionally, reference lists of included articles were reviewed and forward citation searches performed to identify relevant studies. Complete search strategies for each database are presented in the Supplementary Materials (Figure S1).

Following deduplication using EndNote and Rayyan, two authors (H.T. and R.A.) independently screened titles and abstracts from the initial search, with H.T. screening the updated search results. Full text review was performed by H.T. and R.A., with relevant information from non-English language publications translated into English where necessary.

Studies were included based on the following criteria: (1) Population: Transition-aged young people (16–25 years); (2) Concept: Mental health conditions treated in CAMHS; (3) Context: Europe and other high-income countries; (4) Study design: Any design; (5) Publication type: Peer-reviewed articles and abstracts with sufficient information; (6) Language: Any language. Final inclusion was determined by whether studies contributed valuable or novel data to the review objectives.

Relevant data from included studies were systematically extracted by H.T. into a standardised Excel spreadsheet, capturing author details, publication year, country, study aims, design, mental health conditions examined, participant characteristics, and key results. Given the broad scope of the review, encompassing a wide range of study topics, populations, designs and methodologies, a formal quality appraisal of individual studies was not conducted. This review was intended to provide a comprehensive mapping and synthesis of the available evidence rather than a weighted assessment of study rigor. Studies were therefore synthesised narratively to highlight key themes, trends and gaps in the literature. We acknowledge that the absence of formal quality assessment limits conclusions about the strength of the evidence base, and findings should be interpreted accordingly.

## Results

### Search results

The initial search retrieved 10,856 references, with an additional 3,980 references identified in the update. Following deduplication, 12,595 remained for title and abstract screening. Full text review was conducted for 392 articles, of which 139 were included in the final review (published since 2019). Ten further articles were identified via citation searching and Google. A PRISMA flow diagram detailing the selection process is provided in the Supplementary Materials 1 (Figure S2).

The search identified numerous relevant European studies. Many articles were associated with the MILESTONE cohort study and the wider MILESTONE project, which covered eight countries (Belgium, Croatia, France, Germany, Ireland, Italy, the Netherlands, and the UK) [8, 16, 20, 26, 27, 44–59]. Other European studies covered Austria [60, 61], Belgium [11, 62–65], Denmark [66, 67], France [68–72], Germany [22, 67, 73–78], Ireland [23], Italy [9, 79–84], the Netherlands [85, 86], Norway [87–89], Portugal [90], Spain [91–96], Sweden [97], Switzerland [7, 98], and the UK [10, 99–109].

Beyond Europe, recent studies have been conducted in Canada [110–124], the USA [18, 125–128] and Australia [129–131].

The MILESTONE study represents the largest prospective European study to date, following 763 CAMHS users across eight countries for 24 months post-baseline [8, 132]. Detailed methodology and cohort characteristics have been published elsewhere [45, 132]. This cohort study formed part of the broader MILESTONE project on healthcare transition [133], generating multiple sub-studies that contributed to this review [1, 3, 16, 27, 52–56, 58].

All empirical studies have been summarised in Table S1 in Supplementary Materials 2. We also identified additional relevant systematic reviews [6, 19, 25, 33, 84, 113, 134–136], two critical reviews [24, 137], a scoping review [138], and a narrative review [139]. Seven study protocols provided information about relevant past, ongoing or planned research [54, 63, 67, 85, 98, 117, 140].

### Likelihood and predictors of transition

Research examining CAMHS transition practices reveals that clinical recommendations for adult service transfer occur for only a proportion of young people, with referrals not always resulting in care continuing in AMHS [33]. Reasons for this discontinuity are varied: young people may disengage voluntarily, decline medication or onward referral, or find that AMHS thresholds are not met despite a CAMHS recommendation [16, 20, 32, 33, 44]. Service-level factors also play a role, including limited AMHS capacity, mismatches between CAMHS referral and AMHS eligibility criteria, and insufficient availability of suitable adult services [3, 6]. In some cases, young people may also not require ongoing specialist support, having stabilised prior to or around the service boundary [8, 46].

Across multiple studies, those with the most severe mental illnesses are prioritised for AMHS transition [36, 40, 44, 46, 66]. In some cases, this has been linked with diagnoses: several retrospective case note analyses suggest that those with more serious mental health problems such as psychosis or bipolar disorder are more likely to transition [36, 40, 66, 92].

Another study focused just on those who had diagnoses which had been previously identified as being less likely to transition to AMHS: anxiety, depressive and neurodevelopmental disorders [44]. Similarly to other literature, young people who were more severely ill were more likely to transition to AMHS. These findings were echoed in the full sample of the MILESTONE longitudinal cohort study [8, 46]. This study found that young people more likely to transition to AMHS had worse self-reported mental health (as measured by the HoNOSCA completed by a trained research assistant via structured interview with young person, incorporating information from parents/carers, clinicians, and medical records [132, 141]), were rated by their clinician as having more severe mental illness, were experiencing suicidal thoughts or had self-harmed, were on medication, and had higher self or parent-reported need for treatment. Although psychotic experiences were common among young people reaching the service boundary, and persistence or an increase of psychotic experiences were associated with poorer mental health outcomes, they were not associated with continuity of care in AMHS [50]. However, young people with persistently high depressive symptoms were more likely to transition to AMHS compared to those with low depressive symptoms and that that young people with persistent high levels of depressive symptoms who access specialist care after discharge from CAMHS could be identified by risk factors for persistent depression [59]. While these findings suggest that illness severity is a key predictor of transition to AMHS regardless of diagnosis, it is important to note that self- or parent-reported need for treatment may not always align with clinical severity or the findings of formal assessment. Such reports may instead reflect subjective perceptions of need, parental anxiety, or differing thresholds for help-seeking.

Young people with attention deficit hyperactivity disorder (ADHD) are at high risk of discontinuity of care when they reach the CAMHS upper age boundary [36, 40, 66, 97, 99, 139], although those with more severe symptoms are more likely to transition to AMHS [44, 95]. Young people with autism spectrum disorder (ASD) are another group who have been identified as being less likely to transition to AMHS [36, 40] or receive health care transition services [142], although if they have other severe mental health problems they are more likely to transition to AMHS [44].

Rates of transition to AMHS for young people in inpatient services have received comparatively little research attention. In a Danish registry study, transition to AMHS was substantially predicted by inpatient admission [66]. An Italian study explored rates of transition from a child and adolescent inpatient unit and found that around 40% of young people were receiving care at AMHS two years later [81]. This is a higher rate of transition than reported in a different study in Austria, which reported 26% of young people who received care from a child and adolescent psychiatry inpatient unit were later in contact with AMHS inpatient services [15]. However, the composition of cases in the inpatient unit (specifically, the proportion of eating disorders versus other conditions) likely influences the rate of transition to AMHS [19].

In the MILESTONE cohort study, socioeconomic factors did not influence transition recommendations [46]. Although clinical factors appear more influential than socioeconomic factors in determining transition likelihood, a case note review in Northern Ireland - where very few CAMHS referrals were rejected by AMHS - found that young people who were not in employment, education or training (NEET), or had a risk assessment or diagnosis, were more likely to be referred to AMHS [100].

### Service use destinations for young people who reach the upper age boundary of their CAMHS

A systematic review synthesising evidence across studies found that approximately 25% of young people transition to adult mental health services after reaching the CAMHS upper age boundary, with another 25% remaining in CAMHS and 50% having varied service destinations, including discharge to their GP [32]. Disengagement from care was reported in six out of the 11 study cohorts, with rates ranging from 3 to 40% [32]. However, a key limitation of this review is that it was not possible to determine the level of mental health need in the study populations, meaning some young people may not have required continued specialist support after care ending in CAMHS.

Recent European studies have reported similar transition patterns, with few exceptions [100]. A UK retrospective study found that 23% of young people with eating disorders received specialist adult eating disorder care after reaching the upper age boundary of their CAMHS [19, 103], with less than half of these - usually with anorexia nervosa - receiving longer or more intensive treatment. A German study of 32 young people with eating disorders found that only one-third transitioned to AMHS, with nearly 60% remaining in CAMHS up to three years post-boundary [77]. A Spanish retrospective study of 760 young people in Spain reported 29% transitioning to AMHS and 10% staying in CAMHS [92]. Notably for 56% of participants care ended before reaching the age boundary, with dropout being higher in subjects without pharmacological treatment and in those without a recorded diagnosis [92]. Conversely, another Spanish study found 60% of 28 participants transitioned to AMHS, though only one received recommended parallel care (i.e. receiving care from both CAMHS and AMHS around the time of transition) [29]. While showing higher transition rates, the small sample size limits generalisability.

A French retrospective study of 182 young people (mean age 18.1 years) attending CAMHS (*n* = 125) or AMHS (*n* = 57), found only 11.3% of CAMHS patients were considered for adult psychiatry referral, with one-third of young people preferring to remain in CAMHS [72]. Among AMHS patients, 27.7% had been referred from CAMHS.

The MILESTONE longitudinal study of 763 young people identified similar patterns: around 20% transitioned to AMHS and just over 25% remained in CAMHS after reaching service boundaries [8]. A sub-analysis of the data, which focused only on young people with anxiety or mood disorders, neurodevelopmental disorders, or emerging personality disorders, found that those with the most severe illness were more likely to transition to AMHS [44]. However, in Italy, the Netherlands, and the UK, young people with these diagnoses were more likely to experience discontinued care rather than transition, suggesting variation in service provision, eligibility criteria, and availability of CAMHS and AMHS [44].

Significantly, 13% of the full MILESTONE sample were not attending any kind of mental health service at 9-month follow-up but had re-engaged by 15 months (7.8%) or 24 months (5.2%), suggesting inappropriate discharge and care discontinuity [8]. No mental health indicators or sociodemographic characteristics could differentiate these potentially vulnerable young people from others [8]. The pattern of multiple service transitions indicates young people identifying continued care need and searching for suitable mental health support [20, 32, 72].

The MILESTONE cohort study showed that a clinician’s decision or recommendation regarding continuity of treatment, not actual service use outcome, was mostly determined by clinician-rated severity of psychopathology measured with Clinical Global Impression – Severity scale (CGI-S) [46]. A young person or parent-reported need for ongoing treatment increased the odds of clinician recommendation of treatment continuity by more than 2. The recommendation to continue treatment in AMHS, rather than CAMHS, was influenced by service-use related characteristics, such as psychotropic medication use, CAMHS use of over one year, and the availability of suitable AMHS [46]. However, in contrast to previous research findings [36, 40, 143, 144], clinical classification of a severe mental disorder (i.e., bipolar, personality, and schizophrenia spectrum disorders), suicidal thoughts/behaviours or self-harm and psychotic experiences were not positively associated with a recommendation for continuity of care in AMHS. The unexpected findings may be linked to multicollinearity or suppression effect in multivariate analysis [46]. The lack of association with suicidal thoughts/behaviours and self-harm was found to be associated with clinician’s and parent’s unawareness of a young person’s self-reported self-harm and suicidal behaviours [47]. If the clinician was aware of these problems the young person was more likely to transition to AMHS or stay in CAMHS [8, 47]. These findings underline the importance of a standardised assessment regarding self and parent/carer-related problems, including regarding self-harm and suicidal behaviour.

Some young people in Europe can remain at CAMHS beyond the agreed upper age limit, depending on individual services and health service funding structures. The MILESTONE study identified that young people who reported self-harm or suicidal thoughts or behaviours were more likely to stay in CAMHS, suggesting these individuals may have been considered too unstable for transfer [46]. Additionally, CAMHS often continue to work closely with parents and families, which can be particularly important for those with ongoing risk or complex needs. However, extended CAMHS care presents several limitations: not all young people want to remain after reaching the age boundary, not all CAMHS clinicians have appropriate training to support adults over 18 [25], and many countries cannot accommodate this approach due to resourcing, commissioning and legal constraints including medical insurance requirements [1, 56].

For young people with ADHD, a UK surveillance study revealed that 64% of 315 individuals eligible for transition were accepted by AMHS, but only 22% attended their first appointment [99]. Some patients disengaged from services or did not want medication or referral. Since young people with ADHD and other neurodevelopmental disorders are less likely to access AMHS, they can experience difficulties in accessing appropriate support after the service boundary [66, 82, 134, 145], and may be offered or seek out private psychiatrists or psychologists [9], or in some places, continue to be treated in CAMHS [9]. An Italian study, with participating CAMHS from different parts of the country, highlighted that transition trajectories of young people with ADHD varied by CAMHS, potentially reflecting differences in the networks and available resources of the services [82].

### Mental health outcomes for young people transitioning to AMHS, staying in CAMHS and for those whose care is discontinued

There remains a paucity of evidence regarding the longer-term mental health outcomes of young people who reach the CAMHS upper age boundary [32]. The MILESTONE cohort study aimed to address this, however findings should be interpreted with caution given potential selection bias, as only 45.1% of eligible young people (763/1692) were recruited and 16.3% were deemed too unwell by their clinicians to be invited to participate [8]. Over the 24-month follow-up period, the majority of young people showed improvements in their mental health [8]. However, 24.4% of 763 young people had increasing self-reported problems, and 5.3% had a clinically relevant deterioration in their mental health problems, highlighting the need for clear pathways back into mental health services when required [8]. The exclusion of the most vulnerable young people from the study sample suggests these deterioration rates may represent a conservative estimate.

Over the follow-up period, the mental health problems of young people who remained in CAMHS and those who returned to care after discharge did not differ significantly from the mental health problems of young people who transitioned to AMHS [8].

Furthermore, around 50% of young people in MILESTONE, with varying diagnoses and illness severity, stopped using mental health services after reaching the CAMHS upper age boundary. Over the 24-month follow-up period, most of these young people maintained stable mental health and quality of life without ongoing specialist input. However, one quarter (25%) experienced deterioration in their mental health, and 13% required readmission within two years [8, 46]. Young people whose care ended had fewer research assistant-rated and self-reported problems at baseline than those who transitioned to AMHS or remained in CAMHS [46]. Possible reasons for lack of deterioration in their mental health over time include the episodic nature of certain mental health conditions such as depression, natural symptom reduction over time independent of treatment [146], the impact of prior therapeutic interventions, and importantly, young people’s increasing ability to shape their environments. As young adults gain autonomy, they can select educational paths, occupations, and social relationships that align with their individual strengths and challenges, demonstrating positive adaptation [138]. Although about two thirds of young people and parents in the MILESTONE cohort reported a need for ongoing treatment at the service boundary, findings regarding the mental health of those discharged indicate that not all young people who reach the CAMHS upper age boundary require ongoing specialist mental health support. This finding also raises the question of whether some young people could have been discharged from CAMHS earlier [46].

The consequences of poor transitions can be significant for those with ongoing mental health needs [134], including deterioration in mental health [23]. Young people with ADHD may face particular medication management challenges [20, 97, 134, 147], while those with ASD may experience heightened anxiety about forming new therapeutic relationships [139]. When young people with ongoing mental health needs fall through service gaps, many report deteriorating mental health, including poor medication adherence, which can significantly affect their work and educational outcomes [20, 22, 23, 125].

### Experiences of young people, parents/caregivers and health care professionals regarding transition

#### Young person experiences and views

Young people across multiple studies have reported finding the transition period confusing and feeling inadequately prepared [16, 17, 20, 58, 89, 111, 148]. This unpreparedness is compounded by developmental factors, as young people often experience “the feeling of being of age but not yet mature and still needing support” [135, 149], or being treated like everyone else [89]. These feelings of inadequate preparation often lead to fear, anxiety, and uncertainty about new services [77, 89, 109, 111, 150, 151], particularly when young people feel excluded from transition-related decision-making [109, 111, 152, 153]. Such experiences can result in distrust of the healthcare system and feeling let down by CAMHS [89]. For some young people, transition to AMHS can represent an abrupt ending to the trajectory of greater stability and wellbeing achieved in CAMHS, and adapting to a service ethos of chronic mental illness [23], which may be associated with higher levels of stigma [5].

A major challenge at the CAMHS upper age boundary is leaving established therapeutic relationships to start anew at AMHS [16, 20, 77, 109, 118, 151]. Some young people experience intensified feelings of loss, grief, loneliness, and abandonment, due to the attachments developed with CAMHS staff [135, 148], and lack of parental involvement [118, 154]. Young people must often repeat their history to multiple clinicians [17], while facing fragmented or disjointed care, lack of diagnosis-specific expertise, negative interactions with AMHS staff, and long waiting periods without support [23, 109, 148, 155]. The value placed on therapeutic relationships in CAMHS makes building new trust in AMHS particularly challenging [17, 77, 89, 118]. As a result, some young people decline AMHS transition due to these relationship concerns, along with reported long waiting lists, peer accounts of poor care experiences, or relocation for education [16].

Several factors can complicate transition experiences. Financial barriers emerge in countries where CAMHS is free, but AMHS requires payment [16], such as prescription charges for those 18 and older in the UK. Diagnostic issues can also impact transitions – young people without clear or current diagnoses may worry about AMHS acceptance and treatment pathways [58, 156]. Those with personality disorders often experience poorer transitions and continuity of care [101], while complex needs and comorbidities create additional challenges, including negotiating several appointments [115, 148, 157]. For transgender youth, care providers’ lack of knowledge of trans-specific care, lack of a gender-affirming provider or difficulties identifying such a provider, and concerns about respect of confidentiality are additional barriers to adult mental health care [136].

Young people in foster care or local authority care may feel particularly vulnerable and isolated at transition boundaries [43]. Attachment difficulties, which are prevalent in this population, are likely to amplify distrust of new clinical relationships and may compound the challenges of navigating service transitions [43]. Less is known about transition experiences for those with co-occurring physical health conditions [158–160], though studies on transition readiness in this group show mixed results [114–116].

However, transition experiences are not universally negative. Some young people report smooth transitions between services [16] and appreciate the different approaches and perspectives offered by adult services. In the MILESTONE study, using a bespoke measure to capture transition experiences, the majority of young people were satisfied with the process of transition and care ending, though a third of young people still had negative experiences [49]. The overall satisfaction may reflect the fact that CAMHS users who experienced end of care had relatively few mental health problems and did not require continued treatment. In contrast, young people who transitioned to AMHS were less satisfied with their experiences [49].

#### Caregiver experiences and views

Parents/caregivers in the MILESTONE study reported similarly positive experiences to those of the young people; however, most felt excluded from decisions regarding the end or continuation of care [49]. The transition period can be challenging for parents/caregivers to navigate. Many report feeling frustrated and excluded from both the transition process [20, 21, 71, 134] and decisions about their child’s care in AMHS after leaving CAMHS [87, 161]. They can also struggle with developing new relationships with clinicians in the adult setting if they are involved [135]. These negative experiences often result in emotional distress and anxiety for parents and caregivers [6, 71]. Being excluded from discussions and decisions about treatment options can be especially challenging for parents/caregivers if at the same time they are expected to provide care and oversee medication when the young person is still living at home [20].

Parents/caregivers frequently provide post-CAMHS support, helping their children adjust to new services [71] and access self-help resources and other sources of support [20]. However, some struggle to balance this support with promoting their child’s independence [151]. Some parents must assume full responsibility for coordinating their child’s mental health care after CAMHS discharge, particularly when the young person does not transition to AMHS [20]. This includes navigating mental health services, managing GP appointments, and in some cases, managing medication discontinuation when care access is interrupted [20].

In transitions to specialist eating disorder services, parents/caregivers report overwhelming responsibilities while waiting for adult treatment. These include managing their unstable adolescent, providing meal support, driving to appointments, and dealing with treatment uncertainties [87]. These demands often conflict with their other obligations to other children, work commitments and personal needs.

#### Professional experiences and views

Clinicians report significant challenges in transitioning care between CAMHS and AMHS [6, 90, 126]. European-wide surveys confirm these exist across European countries [3, 56]. Key challenges include:

- differing service thresholds and treatment cultures [6, 42, 88, 99]. CAMHS typically accepts patients based on mental distress and dysfunction, while AMHS requires serious mental illness for admission [42]. The services’ care philosophies also diverge: CAMHS employs a nurturing, protective, and family-inclusive approach, while AMHS emphasises diagnostic criteria, acute care, and individual responsibility [6, 78, 88];
- inter-service mistrust and misunderstanding, poor communication and information sharing [28, 78, 88, 90, 162];
- limited resources for patient-centred care [78];
- inflexible transition timing [149];
- lack of transition protocols [3, 9, 83];
- lack of joint working [163];
- lack of professional self-confidence [88]: adult psychiatrists report low confidence in working with young people and families [28];
- high staff turnover [108]; and.
- lack of services or capacity in AMHS because of lack of resources or long waiting lists [3, 10, 99]. AMHS clinicians often lack expertise in conditions like ADHD, with some services refusing to accept ADHD cases entirely [9, 162].

A recent study of English CAMHS clinicians revealed reluctance to discharge patients due to system pressures [108]. Long waiting lists discourage discharge, as clinicians fear patients may deteriorate and face extended waits for re-access. This creates patient accumulation within CAMHS. In contrast, some European countries, like Germany, allow CAMHS clinicians to continue treatment until patients are ready for discharge [1, 77].

### Facilitators of positive transition experiences

Young people and clinicians have identified several transition success factors; a recent review categorised youth preferences across pre-transition, transition, and post-transition phases [164]. Key elements include timely planning, adequate preparation, youth involvement, information sharing, greater flexibility around age boundaries, gradual progression to AMHS, care continuity, and coordination through key workers or joint service meetings [21, 49, 58, 84].

Young people emphasise the importance of being actively involved in transition decisions, including having a voice in the process [111, 135], and autonomy in choosing their transition destination [16]. This highlights the value they place on empowerment and personalised transition plans [135]. Additionally, young people also stress the need for early, regularly reviewed diagnoses [58].

Peer support can aid transition preparation, facilitate engagement, build trust and create hopefulness among young people, especially in inpatient settings [118, 150]. In peer support interventions, former service users draw on their own experiences to support those with mental health needs or their carers – a role that has historically been voluntary but is increasingly being included in the structure of mental health services [165]. The importance of peer support aligns with findings that feeling safe within trusting and supportive environments represents a necessary component for successful care transitions [135].

For transgender youth, additional considerations include access to gender-affirming adult providers and alignment of gender transition goals with mental health care planning [136].

Young people recognise that parental and caregiver readiness for the end of CAMHS care and ongoing involvement are important, particularly for processing medical information and managing appointments and prescriptions [49, 84]. However, not all young people favour such involvement, viewing it as an interference with their autonomy [61] and parental worry and anxiety at this stage can itself become an additional burden for young people to navigate during an already challenging time [6, 20, 151].

Parents/caregivers have expressed their need to participate in decision-making as part of the care team, noting that inclusion helps address their feelings of insecurity while enhancing their ability to support young people who often continue to rely on parental/caregiver assistance [84, 135].

Clinicians have highlighted additional facilitators: increased service availability, flexible transition ages, advance AMHS notification of referrals [10, 166], joint care [135], and person-centred approaches [137]. They advocate for greater patient autonomy [19], recommending that CAMHS prepare young people to manage their own medications and for independent decision-making in AMHS [6, 126, 137, 167]. Facilitators also include clear statutory processes (especially in forensic settings), engagement with family and community services [149], as well as joint training between the services [135], shared electronic records systems [10], and bespoke transition guidelines or protocols [9, 23, 72, 82, 83, 90].

**Primary care** can play a key role in supporting young people during the transition period. Continuous support from primary care during the transition period has been associated with reduced mental health hospitalisations in young adulthood [123]. When specialist services are unavailable, general practitioners (GPs) often become “by default” [104] providers of mental health support [51]. However, while recognising the importance of their involvement [84], many GPs report feeling unprepared for this role [104], particularly regarding specialist medication prescription to young people [104]. While some GPs are expanding their expertise in youth mental health, especially in private practice, research evidence lags behind this trend.

Young people’s experiences regarding their GP vary significantly [51]. Some report negative encounters due to GPs’ unfamiliarity with their history or available support options [51]. Others have encountered medication access issues when their GP has refused to prescribe it irrespective of CAMHS advice [58]. However, young people with established GP relationships report positive experiences in primary care [51].

### Interventions to improve transitional care, experiences and outcomes

Various interventions can support transitional care and improve transition experiences and outcomes between care settings [35, 164]. While no single intervention or transition model shows clear superior effectiveness [24], combining several approaches can enhance transitional mental health care. The interventions operate at patient, clinician and service levels – individually or in combination – with varying implementation complexity. All levels require attention to optimise transition for young people.

#### Service user level interventions

##### Tracking young people reaching the service boundary, assessing transition readiness and care planning

Early identification and tracking of young people approaching the CAMHS-AMHS boundary supports timely planning [69, 113]. This requires organisation-specific criteria, identification processes and monitoring systems [112]. Data science advances could support early identification of young people needing targeted support to prevent disengagement near transition boundaries. For example, a machine learning model using demographic, clinical, and service use data was able to accurately identify 80% of transition-aged young people likely to discontinue mental health services within 12 months [144].

Transition readiness assessment evaluates the knowledge and self-management skills needed to function within adult health care systems [61, 110, 125]. When initiated early, these assessments can be repeated, particularly when readiness or post-transition destinations remain uncertain. Comprehensive assessment should cover mental and physical health, educational and vocational needs, housing requirements, social support systems, practical skills (appointment management, medication adherence), and individual strengths [113].

The Transition Readiness and Appropriateness Measure (TRAM) was developed and validated specifically for mental health services across eight European countries to assess transition readiness and identify barriers that clinicians can address to support smooth transitions [54, 55, 61]. TRAM is a multi-item rating scale comprising 64 items organised into domains covering both the appropriateness of transition and the young person’s readiness for it. It is self-reported across three perspectives, with separate versions completed by the young person, parent/carer, and clinician. Response options vary depending on the question, and are not uniformly Likert in format. The accompanying Transition Related Outcome Measure (TROM) evaluates changes in health care needs post-transition. Both TRAM and TROM [54, 55] cover eight domains: symptoms, overall illness severity, overall life disruption, risk factors, factors affecting symptoms, health system factors, functioning barriers, and other life changes [55]. Comparing self- and parent/caregiver-reports with clinician perspectives identifies knowledge gaps and enhances clinician awareness.

The TRAM and TROM were completed by participants in the MILESTONE study. The study analysis revealed that over half of clinicians and parents were unaware of young people’s self-reported suicidal behaviour at baseline and nine-month follow-up, with this unawareness reducing the likelihood of clinicians recommending continued adult mental health care [47]. In a separate Austrian study where young people completed the TRAM, nearly 80% reported needing continued care, with main transition barriers identified as reluctance to involve parents, difficulty building relationships with the adult care team and problems recounting medical history [61].

The Transition Readiness Assessment Questionnaire (TRAQ) is a 20-item, disease-nonspecific self-report scale validated for mental health services, covering key domains such as medication management, appointment adherence, health monitoring, provider communication, and daily activities [110, 116]. A recent validation study found the updated TRAQ 6.0 version (which excludes daily activities domain) to support CAMHS to AMHS transitions [113]. However, research with young people with co-occurring conditions found the generic scale failed to capture complex needs, requiring in-depth conversations alongside the tool [114].

Transition planning involves multiple actors and should include the young person and appropriate caregivers, with professionals such as psychiatrists, social workers, psychologists, nurses, and occupational therapists [3, 35]. A Delphi study identified seven activities associated with best-practice transition planning: identifying appropriate adult services and determining transfer timing; maintaining updated transition plans; designating a main contact person; initiating planning with adult providers, including joint meetings at least six months prior to transition; communicating with primary care; providing developmentally appropriate resources; and facilitating peer support connections [112].

The Managed Transition model integrates these elements, incorporating transition tracking and TRAM completion by all three perspectives six months prior to the service boundary [133]. Clinicians receive a TRAM findings report displaying responses in graphs and tables to identify critical scores and discrepancies between informants, the findings of which are then discussed with the young person and parent/caregiver at face-to-face appointment to support joint planning and decision-making, and to facilitate adequate communication with the adult care provider [57]. All information and decisions are recorded in individualised transitional care plans developed collaboratively [112, 168].

Managed Transition was tested across eight European countries in a cluster-randomised trial linked to MILESTONE, showing improved mental health and wellbeing in intervention groups compared to usual care controls [57, 132]. Additionally, the use of Managed Transition led to a higher proportion of young people transitioning to AMHS compared to usual care, with the subset of young people with a ‘transition requirement’ being twice as likely to transition to AMHS in the Managed Transition group compared to the group in usual care [169]. Economic evaluation found Managed Transition far less resource intensive and costly than alternative transition programmes [57].

“Got Transition”, an American federally funded national resource centre, provides tools supporting their Six Core Elements of Health Care Transition TM process, offering templates and guides for paediatric to adult health care transitions across all conditions (https://www.gottransition.org/6ce/?leaving-full-package). Evidence shows that this approach has been successfully implemented across diverse clinical settings, supporting structured transition processes and early improvement efforts [170].

##### Education and peer-support

Young people and parents/caregivers would like to receive educational resources covering the transition process and challenges, condition-specific psychoeducation, adult services, and strategies to foster autonomy development [120, 127, 148]. These resources could be personalised with treatment summaries, follow-up appointments, and community/emergency mental health contact details [120].

An example of a co-designed multicomponent transition intervention for young people with eating disorders offers five optional components completed in any order: youth-to-youth group session, parent-to-parent group session, transition meeting with paediatric therapist, family doctor meeting, and a digital navigation guide [121]. In an evaluation study with nine young person-parent pairs and one individual, participants completed 75% of components within three months. While finding the intervention convenient and helpful, with parents reporting decreased guilt, no young people started specialist adult treatment, though three accessed interim supports [121]. Peer support is consistently recommended by service users [148, 150] and integrated in another co-designed intervention, “Moving Forward intervention” (MFi), which targets young people within secure hospitals, and currently awaits full-scale evaluation [118].

##### Digital communication and technological advancements

COVID-19 accelerated digital care adoption, with mental health care professionals continuing with a blended approach, combining digital tools with face-to-face care [171]. Since inaccessible services and poor communication are key transition barriers, digital communications via texts, emails, or video consultations may address some of these problems and improve continuity when face-to-face contact is limited [102, 124].

A qualitative study found that digital communication (text, email and mobile-phone calls) facilitated CAMHS-AMHS transitions by promoting trust and familiarity between staff and service users, fostering greater responsibility in young people and improving access without requiring face-to-face contact [102]. A Delphi study found that young people value planning-focused text interventions (reminders, upcoming dates, actions) more than knowledge-gathering information [124].

Young people with co-occurring chronic health and mental health conditions have described using apps and symptom trackers to manage symptoms, medication, and appointments [115]. Given widespread smartphone ownership, this approach offers significant potential for accessible, cost-effective, self-management support, for example, while waiting to access AMHS, though further research on benefits and disadvantages is needed [115, 172].

Barriers to digital communication - such as limited access to technology and internet, concerns about privacy, insufficient training of health care professionals, and lack of resources - need to be considered when developing digital solutions [172–174].

##### Managed discharge from specialist services

Most young people reaching CAMHS’ upper age boundary do not transition to AMHS but join waiting lists or return to primary care [8, 58]. This requires greater emphasis on managed discharge and interventions supporting self-management without specialist input [108].

Discharge planning is essential for CAMHS transitions [113], yet evidence on effective discharge interventions remains limited. This is particularly critical for inpatient discharge at service boundaries, requiring established protocols [175, 176]. The post-discharge period represents vulnerability, with poor practices and care discontinuity heightening readmission risks [177]. Core elements include thorough assessment, care planning, psychoeducation and self-management support, primary care coordination, community connections, crisis planning, follow-up monitoring, family involvement, and facilitating re-entry when needed [76, 113, 175, 176].

##### Personal health budgets

Personal health budgets provide service users with funds for activities or equipment to support their health and wellbeing, promoting person-centered, recovery-oriented care [178]. While primarily used among adults [178, 179], they have been piloted to improve young people’s discharge from CAMHS. One study funded activities such as music lessons and art equipment, with all participating young people showing improvements on mental health and wellbeing measures following personalised care planning with a dedicated youth facilitator [180]. Although representing only one small-scale study, this indicates the potential of personal health budgets and comprehensive discharge planning.

#### Targeting clinicians and services

##### Transition teams, bespoke coordinators and shared management

Healthcare providers need resources to support transition preparation and care coordination. Multidisciplinary teams with broad service knowledge can address young people’s diverse needs at the service boundaries, enabling smoother transitions while reducing duplication and improving appointment coordination [35].

Dedicated Transition workers – often nurses - are employed to support young people and families through the transition process, ensuring continuity of care [35, 41] and facilitating joint CAMHS-AMHS appointments. A review has identified these transition-focused positions as key to well-developed, structured transition programmes [35]. In the UK, designated transition workers have been successfully embedded, with posts split between AMHS and CAMHS to ensure continuity [39]. In Canada, shared management models with individualised transitional care plans have similarly shown promise [181].

Navigation services and related solutions, e.g., peer navigation and navigation guides, have been implemented in the USA and Canada and help patients navigate complex healthcare systems. They have been viewed favourably by young people, parents/caregivers and professionals in supporting mental health care transitions [119, 121, 122, 127, 128, 182].

Shared management can also occur without dedicated coordinators through transition clinics attended by CAMHS, AMHS, and other relevant professionals [129]. A UK service streamlined its ADHD transition pathways using a Lean approach with joint clinics involving both child/adolescent and adult services, resulting in reducing patients on transfer waiting lists from 134 to 14 and a decrease in waiting times from 12 to four months over 12 months [183].

During fiscal constraints, creating new dedicated transition worker posts can be difficult and they may be lost during restructuring. These positions can also blur clinical responsibilities, fragment care practices, and divide team loyalties [39]. Identifying existing care team members to take on transition coordination responsibilities may offer a more sustainable solution [69, 184].

##### Transition policies, guidelines and service specific protocols

The development and implementation of transition policies, protocols, and reciprocal working arrangements between CAMHS and AMHS provides the foundation for transition-related procedures and practices [75, 112]. Policies are formalised requirements that outline an organisation’s position and values on transition, enhancing organisational readiness [113], while guidelines provide non-mandatory principles of good transitional care, and protocols offer service-specific detailed procedures for defined situations.

Key recommendations across existing relevant guidelines include (1) timely, developmentally appropriate, transition planning and early identification of transition needs, (2) young person and family involvement, (3) information and education provision, (4) care coordination and collaboration between services, (5) clear protocols, pathways and documentation, (6) transition readiness assessment, and (7) addressing holistic needs [29, 34, 117, 184, 185].

Research from Northern Ireland demonstrates that service-specific transition protocols improved transitions [100]. However, the absence of regional policies has led to variability in care delivery, even between teams within the same provider. Research in England, one of the few countries in Europe with national transition policies and guidelines, identified a policy-practice gap [37, 39, 40, 42]. While guidelines can reduce variation in care quality and minimise potential harms [186], they alone cannot resolve longstanding service boundary issues. Clinicians may also struggle to follow guidelines due to workload and resources [163].

Successful implementation requires support for clinicians in improving processes, identification of implementation barriers, and regular review and updates with clinician and service user input [6, 112], using validated tools to evaluate transition experiences of young people and their parents/caregivers [49]. A Delphi expert panel identified essential organisational policy components [112]: (1) Integrated pathway describing transition steps; (2) organisation-specific transition policy developed with service users; (3) youth-centred protocol with communication standards, developed jointly by CAMHS and AMHS; (4) staff training in transition-related skills; (5) clear role definition for all involved (including young person); (6) partnership with the young person and caregivers throughout; and (7) transition protocol evaluation plan.

##### Youth-friendly services

Service restructuring to develop youth-friendly mental health services - typically targeting young people aged 12–25 - aims to eliminate transition barriers at age 18 by reducing help-seeking and engagement obstacles. However, simply raising the transition age fails to resolve other service boundary problems. For example, many of these services, e.g., Maison des Adolescents in France, are aimed at young people with recent onset and low severity symptoms and will refer to CAMHS or AMHS if the young person needs multidisciplinary or longer-term care [68]. This emphasises the need for youth-friendly approaches that can enhance existing services or facilitate smoother transitions.

Approaches implemented by youth-friendly services (Table 1 – Examples of youth friendly services) typically include walk-in sessions, self-referral, designated youth spaces, and integrated “one-stop shop” partnerships that combine health, social care, and voluntary services [187, 188]. Services are usually accessible by public transport and many incorporate peer support. A Belgian Delphi study identified highly relevant, feasible features: free WiFi, pleasant entrance/waiting areas, cozy communal spaces, and quiet rooms [65].

**Table 1:** Examples of youth friendly mental health services

European models • Ireland: Jigsaw [191], • France: Maison des Adolescents (100 + services for ages 11–21) focusing on early-onset, lower-severity symptoms [68], Early detection and intervention centres [70], • the Netherlands: @ease (11 + services for ages 12–25) [85](see also outcomes of a Youth Mental Health team [86]), • Belgium: developments [62, 192], • Italy: PRIMA [80], • UK: Single youth-friendly services: Norfolk Youth Service, Forward Thinking Birmingham [0–25 years], SOLAR, Early Support Hubs in England [107, 193–195]. Other examples include • Australia: headspace [130] • New Zealand: Youth One Stop Shop [196] • Canada: Foundry and ACCESS Open Minds [192].	

Youth-friendly services demonstrate improved engagement, decreased emergency admissions, and reduced symptoms including self-harm and suicidal ideation [131, 187–190]. A recent evaluation of headspace, an Australian youth mental health initiative, shows positive experiences are associated with better service engagement and quality of life [130]. While most youth friendly services have engaged young people and their families in service design, no single model has yet been established as best practice [187].

##### Professional training

Trainees and psychiatrists, and professional associations in Europe have identified mandatory training in mental health service transitions, also called ‘transition psychiatry’, as an important means to improve transitional care [26, 27, 197]. Professional bodies, such as the German scientific associations of child and adolescent psychiatry and psychotherapy, and adult psychiatry and psychotherapy (DGKJP and DGPPN), have produced position statements regarding mental health service transitions and outlined key measures to improve the processes [197]. They have also identified essential competencies for psychiatrists regarding mental health service transitions to ensure clinicians have the expertise to manage transitions [74]. The focus is on developing specialised knowledge and skills to effectively support young people as they transition between different stages of development and systems of care. These include understanding developmental psychopathology, addressing comorbidities like learning and developmental disorders, involving families, collaborating across relevant systems, using age-appropriate interventions, coordinating individualised care, adapting to changing needs, and maintaining legal and ethical awareness [74]. The overarching goal is to cultivate a flexible, patient-centred, and respectful approach to facilitate successful transitions for young people.

##### Collaboration with primary care

Primary care plays a crucial role in supporting young people who have been discharged from specialist mental health services, no longer wish to attend such services, or are awaiting AMHS appointments. Given the significant gaps that can occur in specialist care, strengthening primary care capacity through various collaborative approaches has emerged as a key strategy to ensure continuity of mental health support during transition periods [198]. This is particularly relevant for ADHD care, where not all patients need specialist mental health services - many can be well-managed by GPs in shared care arrangements, especially for straightforward cases without other mental health conditions [104, 105, 199].

Training GPs in prescribing psychotropic medication, effective youth communication and transition challenges may enhance their confidence in managing care for these young people and improve outcomes [200, 201]. Making every contact count, while acknowledging that there is no health without mental health helps build rapport and communication between GPs and young people [200].

Primary care providers typically welcome mental health care capacity building, though many programmes lack rigorous evaluation [202]. Mental health nurses in primary care can help support young people who are no longer attending specialist services, improving outcomes, satisfaction, access times and reducing stigma [203, 204].

Collaborative care models could facilitate smoother transitions from CAMHS to primary care by providing structured, proactive management of young people’s ongoing mental health needs [201]. This approach involves a multidisciplinary team – comprising the GP, a mental health practitioner (such as a psychological wellbeing practitioner or mental health nurse), and consulting psychiatrist – who together coordinate regular follow-up and monitoring, rather than solely responding to acute presentations. Collaborative care has demonstrated effectiveness in treating common mental health problems in adult populations [205]. However, such models remain underexplored in youth mental health [106, 201].

## Discussion

This review synthesises evidence on care transitions at CAMHS upper age boundaries to inform clinical guidance development. The findings reveal significant variations in transition patterns and outcomes across Europe, while identifying key evidence-based factors that influence transition experiences and mental health trajectories.

### The evidence base: understanding transition patterns and outcomes

International research demonstrates that transition to adult services occurs in only 20–25% of young people who reach the upper age boundary of their CAMHS, with those experiencing severe mental illness prioritised regardless of specific diagnosis [44]. This challenges earlier assumptions that continuity of care was typically warranted only for certain diagnostic groups, particularly those with severe disorders like psychosis [40, 143, 144]. Across multiple studies and countries, successful transition to AMHS occurs consistently in approximately one-quarter of young people who reach the CAMHS upper age boundary, though significant country and service-specific variations exist, with clinicians’ recommendations often influenced by the availability of suitable adult services [1, 3, 8].

The majority of young people (40–60%) discontinue specialist mental health services at CAMHS upper age boundaries for varied reasons [32, 33]. However, follow-up data from the MILESTONE study reveals that most maintain stable mental health over 24 months, suggesting that specialist input is no longer required for many [8]. This group may include those whose conditions naturally fluctuate (such as depression) or those who cannot access services due to relocation for education [152]. For those who do maintain stable mental health, discharge from specialist services can facilitate the development of personal agency and coping skills to manage their wellbeing independently [206].

However, these aggregate figures mask important variations, and may underestimate deterioration rates due to selection bias toward less severely ill participants [8, 46]. A substantial minority (25%) of MILESTONE study participants experienced deteriorating mental health post-transition, with 13% requiring readmission within two years. These findings highlight the vulnerability of a significant subgroup, indicating system failures in transition for some cases and the importance of managed discharge alongside transition. The consequences of poor mental health outcomes at this critical life stage are substantial, with young people aged 16–25 with mental health problems significantly more likely to be not in employment, education or training (27% vs. 16%) [207]. Recognising young people at risk of worsening depressive symptoms before they leave CAMHS may support smoother transitions and improve outcomes, including continuity of care [59].

Notably, several European countries demonstrate flexibility in CAMHS age boundaries, with some young people continuing to receive care beyond standard limits [10, 59, 108]. The reasons for this extended care - whether reflecting beneficial treatment consolidation that obviates AMHS need, clinical judgment that service changes pose excessive risk, or simply limited AMHS availability forcing extended CAMHS care – require clarification [1, 46, 56]. Complex cases involving trauma, adverse life experiences, and challenging family dynamics may further contribute to clinicians’ reluctance to discharge, particularly where re-access is difficult [126].

### Critical factors influencing transition success

Research across Europe demonstrates that poor transition experiences are common, especially for young people requiring continuity of care. Only few young people experience optimal transitions – characterised by adequate transition planning, good information transfer across teams, joint working between teams and continuity of care following transition [40]. However, the evidence identifies several modifiable factors associated with improved transition experiences and outcomes.

#### Involvement and preparation of young people and parents/caregivers in transition planning

Involving young people and parents/caregivers from the start in transition planning emerges consistently as a key factor in successful transitions or positive experiences. Despite this, young people report exclusion from decision-making and inadequate preparation, causing confusion and anxiety about new services. However, some report smooth transitions and appreciate adult services’ approaches. The MILESTONE study found that a majority of participants were satisfied with transition processes, though a third had negative experiences, with higher satisfaction among those ending care than transitioning to AMHS [49]. Caregivers feel similarly excluded, with some parents assuming full responsibility for managing their child’s mental health care when AMHS transition fails.

The evidence suggests that preparation for independent care management should begin while young people remain in CAMHS, particularly given that discharge to primary care is more common than transition to AMHS. This preparation requires young people to be equipped with comprehensive information about their condition, diagnosis, and local support structures. However, not all young people desire parental involvement in their care decisions, and this preference should be respected.

#### Systematic assessment and planning approaches

Structured assessment tools such as the TRAM facilitate discussions among stakeholders about available options and preparation strategies. Those tools are intended to guide – not determine – service allocation. Research suggests optimal implementation involves initial assessment approximately six months before the service boundary, with potential reassessment closer to transition to capture changes in symptoms and circumstances. The effectiveness of this structured approach is demonstrated by the Managed Transition intervention utilising the TRAM, which showed improved outcomes and cost-effectiveness compared to usual care across eight European countries [52, 57, 169].

#### Service system factors

Clinicians identify substantial barriers including differing thresholds and treatment cultures between services. AMHS availability significantly influences transition decisions, particularly in countries where service age boundaries are adhered to [44, 82], creating situations where clinical decisions are constrained by resource limitations rather than clinical appropriateness. These varied transition patterns are also associated with varied costs [44, 52]. Continued specialist mental health care after the service boundary accrues the most healthcare costs; however, the cost of unmet need for those unable to access services may manifest in long-term costs and potential worse health outcomes [52].

#### Emerging supportive interventions

The evidence for peer support as a facilitating factor in mental health care transitions reflects broader research demonstrating its effectiveness across various mental health contexts. Peer support interventions have been shown to reduce psychiatric symptoms, improve social functioning, and enhance service engagement among young adults with mental health conditions [208, 209]. The mechanism appears to operate through shared lived experience, which can reduce stigma and provide credible hope for recovery. In transition, peer support is relevant for both young people and their parents/caregivers.

Primary care involvement shows promise but remains under-researched. Limited evidence suggests that proactive primary care engagement can facilitate transitions and provide essential follow-up support, potentially supporting the development of personal agency while enabling early detection of mental health deterioration to prevent readmission. However, further research is needed to establish optimal models for primary care involvement in mental health transitions [201].

### Implications for evidence-based guidance development

The evidence reveals substantial heterogeneity in young people’s transition needs and optimal pathways. Young people requiring particular attention are those who may not meet AMHS thresholds but whose mental health deteriorates either while remaining in CAMHS or after discharge [72]. This group includes young people with self-harm or suicidal behaviours that may be unknown to clinicians and parents during transition planning [47]. This variation necessitates individualised approaches within systematic frameworks, informing guidance that balances standardisation with flexibility.

Earlier conceptualisations of optimal transition focused primarily on systemic factors such as transition planning, information transfer, joint working, and care continuity [40]. While these remain important, current evidence expands understanding of optimal transition to include youth autonomy, appropriate parental engagement where desired, and peer support as critical elements [53]. This evolution reflects growing recognition of young people’s autonomy and the complex social context of transition experiences.

Previous transition interventions have typically been developed without significant service user input [5], and have been more service oriented [164]. In contrast, co-production has become central to recently developed transition interventions [117, 118, 140, 148], representing best practice that should be maintained despite more complex and lengthy development processes involved [106]. Co-production ensures interventions address the actual needs and preferences of young people and families, potentially improving both acceptability and effectiveness of transition support [210].

Given that AMHS availability significantly influences transition decisions, governments must ensure adequate adult service provision with eligibility criteria that meet ongoing treatment needs of young people reaching CAMHS boundaries. Without appropriate adult services, transitions become constrained by resource limitations rather than clinical appropriateness. Greater emphasis should be placed on identifying those at risk of deterioration, such as via Managed Transition, and improving transitions to primary care – including proactive follow-up in the period immediately following discharge – to prevent future readmissions to specialist services.

### Evidence gaps and research implications

#### Study limitations affecting guidance development

The review covers the latest European literature on mental health care transitions, including publications from the MILESTONE study which is the largest prospective study conducted so far on mental health outcomes and service destinations for young people who have reached the upper age boundary of their CAMHS [8, 16, 44–46, 49, 50, 52, 59]. However, due to the extensive nature of the review, we did not assess the quality of the included studies.

Many findings derive mainly from MILESTONE, examining transitions across the child-adult mental health service boundary in eight countries. Further longitudinal studies are needed to confirm these findings. A limitation of the MILESTONE cohort relates to the generalisability of its findings. Whilst young people in care were represented in the cohort – including those with adoptive/foster parents (2.1%) and those in residential care (3.5%) – individuals in secure forensic institutions or with an intellectual disability (IQ < 70) were excluded [45]. Given that young people in care are known to be overrepresented in CAMHS caseloads relative to the general population [211], it is possible that these groups remain underrepresented in the cohort. Combined with the selection bias inherent in the cohort [45], the current evidence may understate negative outcomes, particularly for young people with the most complex needs, who are often underrepresented in research participation.

However, the findings can be generalised independent of service type or European country since participating young people were from a wide range of CAMHS in different European countries, varying in size and ranging from community to specialist and/or hospital-based services in countries with differences in mental health service organisation [46].

#### Priority areas for future research

While the number of European studies on transition has increased significantly over the past six years, with a greater number of countries represented, most of the studies are relatively small scale and/or retrospective in nature. Future research should prioritise representative longitudinal studies that capture longer-term outcomes for the most vulnerable young people at CAMHS transition boundaries. This includes developing ethical frameworks and methodological approaches that enable inclusion of young people who are acutely unwell, while ensuring appropriate safeguards. Studies employing alternative recruitment strategies, such as registry-based cohorts or routine clinical data linkage, could provide more comprehensive population-level insights into post-transition mental health trajectories [212, 213]. Additionally, research is needed to understand the specific needs and outcomes of young people excluded from current studies, potentially through targeted follow-up studies or healthcare utilisation data analysis, and to establish optimal models for primary care involvement in transitions. Studies focusing on the quality of care in CAMHS and AMHS and associated transition and mental health outcomes are also needed.

### Conclusion - contextualising the clinical guidance

This evidence synthesis reveals that while poor transition experiences remain common across Europe, several modifiable factors can significantly improve outcomes. Importantly, the research demonstrates that improvements in transitional care can positively impact mental health outcomes regardless of final service destination [57]. This underscores the influential role of individual clinicians, who – within existing organisational constraints – can improve the quality of transitional care through structured and timely preparation, meaningful stakeholder involvement, and continuity of support across service boundaries.

The evidence challenges deficit-focused perspectives on service ending, instead highlighting that service discontinuation at CAMHS boundaries represents the appropriate pathway for approximately half of young people, with many maintaining stable mental health over subsequent years. This finding highlights the importance of supporting young people’s development of personal agency and independent coping skills rather than viewing all service endings as system failures.

However, the substantial variations in transition patterns across countries and services, often driven by resource availability rather than clinical need, underscore the importance of both individual clinical approaches and system-level service development. The clinical guidance must therefore address both immediate clinical decision-making and longer-term service improvement, emphasising the need for individualised, developmentally appropriate approaches that respect young people’s autonomy while providing necessary support during this critical life stage.

These evidence-based insights directly informed the development of the ESCAP Transition Guidance (see companion guidance article [30]), ensuring that the recommendations address both individual clinical practice and systemic service improvements highlighted by this review.

## Supplementary Information

Below is the link to the electronic supplementary material.

Supplementary Material 1

Supplementary Material 2

## Data Availability

No datasets were generated or analysed during the current study.
