# Supplementary material for: Transitions from child to adult mental health care: the evidence-base for ESCAP guidance for clinicians
Source: Eur Child Adolesc Psychiatry. 2026 Apr 25;35(8):2569–91. doi: 10.1007/s00787-026-03036-0 (PMC13652868; doi:10.1007/s00787-026-03036-0)
Supplement: Supplementary file 1 [file 787_2026_3036_MOESM1_ESM.docx]

Transitions from child to adult mental health care: the evidence-base for ESCAP guidance for clinicians

Supplement

Helena Tuomainen^1^, Rebecca Appleton^2^, Gwendolyn C. Dieleman^3^, Tomislav Franić^4^, Giovanni de Girolamo^5^, Athanasios Maras^6^, Fiona McNicholas^7, 8, 9^, Mathilde M. Overbeek^6, 10, 11^, Diane Purper-Ouakil^12, 13^, Paramala Santosh^14, 15, 16^, Ulrike M.E. Schulze^17^, Cathy Street^1^, Dieter Wolke^18^, Swaran P. Singh^1^

**Corresponding author**

Dr Helena Tuomainen

Warwick Medical School

University of Warwick

Coventry CV47AL

helena.tuomainen@warwick.ac.uk

## Figure S1. Database searches

### Searches 30^th^ August 2023

Embase – 30^th^ August 2023

6136 total

Embase Classic+Embase <1947 to 2023 Week 34>

1	Transition to Adult Care/	2905

2	exp "Continuity of Patient Care"/	1028432

3	Transitional Care/	5543

4	(transition* or transfer* or continuity).ab.	1451244

5	(transition* or transfer* or continuity).ti.	304083

6	((transition* or transfer* or continuity or interface) adj5 care).mp.	58354

7	continuity of care.mp.	12699

8	exp Critical Pathways/	9924

9	care pathway.mp.	7749

10	1 or 2 or 3 or 6 or 7 or 8 or 9	1073811

11	mental health services.mp. or exp Mental Health Services/	78903

12	("mental health" adj5 service*).mp. [mp=title, abstract, heading word, drug trade name, original title, device manufacturer, drug manufacturer, device trade name, keyword heading word, floating subheading word, candidate term word]	86292

13	camhs.mp.	1129

14	amhs.mp.	262

15	mental health.mp. or exp Mental Health/	433969

16	Mental disorders.mp. or exp Mental Disorders/	2806647

17	Psychiatry.mp. or exp Psychiatry/	218113

18	psychiatr*.mp.	515217

19	mental illness*.mp.	55841

20	Neurodevelopmental disorder.mp. or exp Neurodevelopmental Disorders/	2796271

21	Attention deficit hyperactivity disorder.mp. or exp Attention Deficit Disorder with Hyperactivity/	45085

22	ADHD.mp.	46793

23	(Autis* or ASD or ASC).mp. [mp=title, abstract, heading word, drug trade name, original title, device manufacturer, drug manufacturer, device trade name, keyword heading word, floating subheading word, candidate term word]	137398

24	exp Depressive Disorder/ or Depressive disorder.mp.	635278

25	Anxiety disorder.mp. or exp Anxiety Disorders/	329995

26	Personality disorder.mp. or exp Personality Disorders/	77671

27	Eating disorder.mp. or exp "Feeding and Eating Disorders"/	67883

28	11 or 12 or 13 or 14 or 15 or 16 or 17 or 18 or 19 or 20 or 21 or 22 or 23 or 24 or 25 or 26 or 27	3195281

29	Young adult.mp. or exp Young Adult/	546450

30	exp Adolescent/ or Adolescent.mp.	1967683

31	adolescen*.mp.	2042893

32	teenager*.mp.	24954

33	Pediatrics.mp. or exp Pediatrics/	195615

34	pa*diatric*.mp.	151390

35	exp Child/ or Child.mp.	3773228

36	29 or 30 or 31 or 32 or 33 or 34 or 35	5134104

37	10 and 28 and 36	26933

38	limit 37 to yr="2019 -Current"	8849

39	limit 38 to (article or article in press or "preprint (unpublished, non-peer reviewed)" or "review")	6136

<https://ovidsp.ovid.com/ovidweb.cgi?T=JS&NEWS=N&PAGE=main&SHAREDSEARCHID=3H34P1BJ3vM99o1JmfMKvtfxASf3qulYvxjP9Co6nWZigjcM2ErID2sFwTqRv636p>

PsychInfo – 30^th^ Aug 2023

341 total

APA PsycInfo <1806 to August Week 3 2023>

1	Transition to Adult Care/	0

2	exp "Continuity of Patient Care"/	0

3	Transitional Care/	0

4	(transition* or transfer* or continuity).ab.	173024

5	(transition* or transfer* or continuity).ti.	41426

6	((transition* or transfer* or continuity or interface) adj5 care).mp.	10581

7	continuity of care.mp.	2700

8	exp Critical Pathways/	0

9	care pathway.mp.	657

10	1 or 2 or 3 or 6 or 7 or 8 or 9	11192

11	mental health services.mp. or exp Mental Health Services/	75098

12	("mental health" adj5 service*).mp. [mp=title, abstract, heading word, table of contents, key concepts, original title, tests & measures, mesh word]	83083

13	camhs.mp.	809

14	amhs.mp.	83

15	mental health.mp. or exp Mental Health/	284786

16	Mental disorders.mp. or exp Mental Disorders/	1089927

17	Psychiatry.mp. or exp Psychiatry/	112601

18	psychiatr*.mp.	376105

19	mental illness*.mp.	55072

20	Neurodevelopmental disorder.mp. or exp Neurodevelopmental Disorders/	238780

21	Attention deficit hyperactivity disorder.mp. or exp Attention Deficit Disorder with Hyperactivity/	37142

22	ADHD.mp.	34598

23	(Autis* or ASD or ASC).mp. [mp=title, abstract, heading word, table of contents, key concepts, original title, tests & measures, mesh word]	71355

24	exp Depressive Disorder/ or Depressive disorder.mp.	79621

25	Anxiety disorder.mp. or exp Anxiety Disorders/	56067

26	Personality disorder.mp. or exp Personality Disorders/	48819

27	Eating disorder.mp. or exp "Feeding and Eating Disorders"/	19087

28	11 or 12 or 13 or 14 or 15 or 16 or 17 or 18 or 19 or 20 or 21 or 22 or 23 or 24 or 25 or 26 or 27	1397855

29	Young adult.mp. or exp Young Adult/	178885

30	exp Adolescent/ or Adolescent.mp.	427762

31	adolescen*.mp.	521997

32	teenager*.mp.	10541

33	Pediatrics.mp. or exp Pediatrics/	41009

34	pa*diatric*.mp.	6575

35	exp Child/ or Child.mp.	526692

36	29 or 30 or 31 or 32 or 33 or 34 or 35	974191

37	10 and 28 and 36	1219

38	limit 37 to yr="2019 -Current"	341

Medline 30^th^ August 2023

2139 total

Ovid MEDLINE(R) ALL <1946 to August 28, 2023>

1	Transition to Adult Care/	2124

2	exp "Continuity of Patient Care"/	290912

3	Transitional Care/	1256

4	(transition* or transfer* or continuity).ab.	1225840

5	(transition* or transfer* or continuity).ti.	279732

6	((transition* or transfer* or continuity or interface) adj5 care).mp.	50587

7	continuity of care.mp.	9126

8	exp Critical Pathways/	7836

9	care pathway.mp.	4259

10	1 or 2 or 3 or 6 or 7 or 8 or 9	323536

11	mental health services.mp. or exp Mental Health Services/	117456

12	("mental health" adj5 service*).mp. [mp=title, book title, abstract, original title, name of substance word, subject heading word, floating sub-heading word, keyword heading word, organism supplementary concept word, protocol supplementary concept word, rare disease supplementary concept word, unique identifier, synonyms, population supplementary concept word, anatomy supplementary concept word]	75210

13	camhs.mp.	664

14	amhs.mp.	200

15	mental health.mp. or exp Mental Health/	272256

16	Mental disorders.mp. or exp Mental Disorders/	1453585

17	Psychiatry.mp. or exp Psychiatry/	151623

18	psychiatr*.mp.	402347

19	mental illness*.mp.	41179

20	Neurodevelopmental disorder.mp. or exp Neurodevelopmental Disorders/	213931

21	Attention deficit hyperactivity disorder.mp. or exp Attention Deficit Disorder with Hyperactivity/	44794

22	ADHD.mp.	32194

23	(Autis* or ASD or ASC).mp. [mp=title, book title, abstract, original title, name of substance word, subject heading word, floating sub-heading word, keyword heading word, organism supplementary concept word, protocol supplementary concept word, rare disease supplementary concept word, unique identifier, synonyms, population supplementary concept word, anatomy supplementary concept word]	91824

24	exp Depressive Disorder/ or Depressive disorder.mp.	135141

25	Anxiety disorder.mp. or exp Anxiety Disorders/	100724

26	Personality disorder.mp. or exp Personality Disorders/	50576

27	Eating disorder.mp. or exp "Feeding and Eating Disorders"/	40367

28	11 or 12 or 13 or 14 or 15 or 16 or 17 or 18 or 19 or 20 or 21 or 22 or 23 or 24 or 25 or 26 or 27	1882255

29	Young adult.mp. or exp Young Adult/	1039141

30	exp Adolescent/ or Adolescent.mp.	2261271

31	adolescen*.mp.	2311426

32	teenager*.mp.	17042

33	Pediatrics.mp. or exp Pediatrics/	99037

34	pa*diatric*.mp.	86614

35	exp Child/ or Child.mp.	2379041

36	29 or 30 or 31 or 32 or 33 or 34 or 35	4154765

37	10 and 28 and 36	12706

38	limit 37 to yr="2019 -Current"	2319

<https://ovidsp.ovid.com/ovidweb.cgi?T=JS&NEWS=N&PAGE=main&SHAREDSEARCHID=3lUvAkve4l7qlNjrNRtILBzVPEvAqVDzo6jVhQYCb8k0oWOLrZL2cl5MhOYJrHOCA>

Total MEDLINE 2319 – when importing to EndNOte 45 removed as duplicates, leaving 2274

Web of Science 30^th^ august 2023

2060 total

<https://www.webofscience.com/wos/woscc/summary/6cbeaa6f-0fe5-43d3-8478-35e37aaec135-a09fbf14/relevance/1>

### Searches 10th April 2025

Medline

Ovid MEDLINE(R) ALL <1946 to April 09, 2025>

1	Transition to Adult Care/	2504

2	exp "Continuity of Patient Care"/	311001

3	Transitional Care/	1444

4	(transition* or transfer* or continuity).ab.	1358089

5	(transition* or transfer* or continuity).ti.	303606

6	((transition* or transfer* or continuity or interface) adj5 care).mp.	56230

7	continuity of care.mp.	10411

8	exp Critical Pathways/	8284

9	care pathway.mp.	5202

10	1 or 2 or 3 or 6 or 7 or 8 or 9	348708

11	mental health services.mp. or exp Mental Health Services/	124100

12	("mental health" adj5 service*).mp. [mp=title, book title, abstract, original title, name of substance word, subject heading word, floating sub-heading word, keyword heading word, organism supplementary concept word, protocol supplementary concept word, rare disease supplementary concept word, unique identifier, synonyms, population supplementary concept word, anatomy supplementary concept word]	81160

13	camhs.mp.	764

14	amhs.mp.	225

15	mental health.mp. or exp Mental Health/	318387

16	Mental disorders.mp. or exp Mental Disorders/	1557457

17	Psychiatry.mp. or exp Psychiatry/	158953

18	psychiatr*.mp.	432876

19	mental illness*.mp.	45890

20	Neurodevelopmental disorder.mp. or exp Neurodevelopmental Disorders/	228300

21	Attention deficit hyperactivity disorder.mp. or exp Attention Deficit Disorder with Hyperactivity/	49064

22	ADHD.mp.	36244

23	(Autis* or ASD or ASC).mp. [mp=title, book title, abstract, original title, name of substance word, subject heading word, floating sub-heading word, keyword heading word, organism supplementary concept word, protocol supplementary concept word, rare disease supplementary concept word, unique identifier, synonyms, population supplementary concept word, anatomy supplementary concept word]	105324

24	exp Depressive Disorder/ or Depressive disorder.mp.	143402

25	Anxiety disorder.mp. or exp Anxiety Disorders/	108148

26	Personality disorder.mp. or exp Personality Disorders/	52536

27	Eating disorder.mp. or exp "Feeding and Eating Disorders"/	43653

28	11 or 12 or 13 or 14 or 15 or 16 or 17 or 18 or 19 or 20 or 21 or 22 or 23 or 24 or 25 or 26 or 27	2043319

29	Young adult.mp. or exp Young Adult/	1149035

30	exp Adolescent/ or Adolescent.mp.	2368362

31	adolescen*.mp.	2426100

32	teenager*.mp.	18379

33	Pediatrics.mp. or exp Pediatrics/	107829

34	pa*diatric*.mp.	96587

35	exp Child/ or Child.mp.	2500040

36	29 or 30 or 31 or 32 or 33 or 34 or 35	4399601

37	10 and 28 and 36	13714

38	limit 37 to yr="2019 -Current"	3293

39	limit 38 to ed=20230831-20250410	912

40	limit 38 to ep=20230831-20250410	689

41	limit 38 to dt=20230831-20250410	835

42	39 or 40 or 41	**974**

PsychInfo

APA PsycInfo <2002 to March 2025 Week 5>

1	Transition to Adult Care/	0

2	exp "Continuity of Patient Care"/	0

3	Transitional Care/	0

4	(transition* or transfer* or continuity).ab.	141184

5	(transition* or transfer* or continuity).ti.	31079

6	((transition* or transfer* or continuity or interface) adj5 care).mp.	10350

7	continuity of care.mp.	2571

8	exp Critical Pathways/	0

9	care pathway.mp.	785

10	1 or 2 or 3 or 6 or 7 or 8 or 9	11083

11	mental health services.mp. or exp Mental Health Services/	79513

12	("mental health" adj5 service*).mp. [mp=title, abstract, heading word, table of contents, key concepts, original title, tests & measures, mesh word]	65546

13	camhs.mp.	897

14	amhs.mp.	99

15	mental health.mp. or exp Mental Health/	257403

16	Mental disorders.mp. or exp Mental Disorders/	812228

17	Psychiatry.mp. or exp Psychiatry/	78541

18	psychiatr*.mp.	249263

19	mental illness*.mp.	45959

20	Neurodevelopmental disorder.mp. or exp Neurodevelopmental Disorders/	126588

21	Attention deficit hyperactivity disorder.mp. or exp Attention Deficit Disorder with Hyperactivity/	34881

22	ADHD.mp.	33341

23	(Autis* or ASD or ASC).mp. [mp=title, abstract, heading word, table of contents, key concepts, original title, tests & measures, mesh word]	68486

24	exp Depressive Disorder/ or Depressive disorder.mp.	165022

25	Anxiety disorder.mp. or exp Anxiety Disorders/	45801

26	Personality disorder.mp. or exp Personality Disorders/	31442

27	Eating disorder.mp. or exp "Feeding and Eating Disorders"/	18588

28	11 or 12 or 13 or 14 or 15 or 16 or 17 or 18 or 19 or 20 or 21 or 22 or 23 or 24 or 25 or 26 or 27	1042593

29	Young adult.mp. or exp Young Adult/	180753

30	exp Adolescent/ or Adolescent.mp.	333103

31	adolescen*.mp.	392397

32	teenager*.mp.	8556

33	Pediatrics.mp. or exp Pediatrics/	36491

34	pa*diatric*.mp.	6915

35	exp Child/ or Child.mp.	365586

36	29 or 30 or 31 or 32 or 33 or 34 or 35	726764

37	10 and 28 and 36	1261

38	limit 37 to yr="2019 -Current"	514

39	limit 38 to yr="2023 -Current"	158

Embase

Embase <1980 to 2025 Week 14>

1	Transition to Adult Care/	3434

2	exp "Continuity of Patient Care"/	1119811

3	Transitional Care/	6613

4	(transition* or transfer* or continuity).ab.	1533570

5	(transition* or transfer* or continuity).ti.	302070

6	((transition* or transfer* or continuity or interface) adj5 care).mp.	66249

7	continuity of care.mp.	13953

8	exp Critical Pathways/	10843

9	care pathway.mp.	9210

10	1 or 2 or 3 or 6 or 7 or 8 or 9	1170879

11	mental health services.mp. or exp Mental Health Services/	78466

12	("mental health" adj5 service*).mp. [mp=title, abstract, heading word, drug trade name, original title, device manufacturer, drug manufacturer, device trade name, keyword heading word, floating subheading word, candidate term word]	86506

13	camhs.mp.	1381

14	amhs.mp.	291

15	mental health.mp. or exp Mental Health/	482949

16	Mental disorders.mp. or exp Mental Disorders/	2927813

17	Psychiatry.mp. or exp Psychiatry/	196811

18	psychiatr*.mp.	495417

19	mental illness*.mp.	59260

20	Neurodevelopmental disorder.mp. or exp Neurodevelopmental Disorders/	2923143

21	Attention deficit hyperactivity disorder.mp. or exp Attention Deficit Disorder with Hyperactivity/	91385

22	ADHD.mp.	52196

23	(Autis* or ASD or ASC).mp. [mp=title, abstract, heading word, drug trade name, original title, device manufacturer, drug manufacturer, device trade name, keyword heading word, floating subheading word, candidate term word]	153777

24	exp Depressive Disorder/ or Depressive disorder.mp.	707949

25	Anxiety disorder.mp. or exp Anxiety Disorders/	366207

26	Personality disorder.mp. or exp Personality Disorders/	76301

27	Eating disorder.mp. or exp "Feeding and Eating Disorders"/	72186

28	11 or 12 or 13 or 14 or 15 or 16 or 17 or 18 or 19 or 20 or 21 or 22 or 23 or 24 or 25 or 26 or 27	3283479

29	Young adult.mp. or exp Young Adult/	631593

30	exp Adolescent/ or Adolescent.mp.	1917594

31	adolescen*.mp.	1992203

32	teenager*.mp.	25836

33	Pediatrics.mp. or exp Pediatrics/	190348

34	pa*diatric*.mp.	164210

35	exp Child/ or Child.mp.	3424822

36	29 or 30 or 31 or 32 or 33 or 34 or 35	4809556

37	10 and 28 and 36	30868

38	limit 37 to yr="2019 -Current"	13104

39	limit 38 to dd=20230831-20250410	4047

40	limit 38 to dc=20230831-20250410	4349

41	39 or 40	4349

42	limit 41 to "remove preprint records"	4349

43	limit 42 to (article or article in press)	2578

Web of Science

<https://www.webofscience.com/wos/woscc/summary/8548eae9-78c8-4cd2-9398-f29b66de0f23-0159c3fc64/relevance/1>

## Figure S2. Prisma flow diagram

Source: Page MJ, et al. BMJ 2021;372:n71. doi: 10.1136/bmj.n71.

This work is licensed under CC BY 4.0. To view a copy of this license, visit <https://creativecommons.org/licenses/by/4.0/>
