# Supplementary material for: Transitions from child to adult mental health care: the evidence-base for ESCAP guidance for clinicians
Source: Eur Child Adolesc Psychiatry. 2026 Apr 25;35(8):2569–91. doi: 10.1007/s00787-026-03036-0 (PMC13652868; doi:10.1007/s00787-026-03036-0)
Supplement: Supplementary file 2 [file 787_2026_3036_MOESM2_ESM.docx]

**Transitions from child to adult mental health care: the evidence-base for ESCAP guidance for clinicians**

Supplement

Helena Tuomainen^1^, Rebecca Appleton^2^, Gwendolyn C. Dieleman^3^, Tomislav Franić^4^, Giovanni de Girolamo^5^, Athanasios Maras^6^,
Fiona McNicholas^7, 8, 9^, Mathilde M. Overbeek^6, 10, 11^, Diane Purper-Ouakil^12, 13^, Paramala Santosh^14, 15, 16^, Ulrike M.E. Schulze^17^,
Cathy Street^1^, Dieter Wolke^18^, Swaran P. Singh^1^

**Corresponding author**

Helena Tuomainen

Warwick Medical School

University of Warwick

Coventry CV47AL

United Kingdom

helena.tuomainen@warwick.ac.uk

Table S1. Characteristics of included empirical studies published since 2019

| **Author (year)** | **Country** | **Aim** | **Study design** | **Mental health condition** | **Participants - number, age, other characteristics** | **Relevant results** |
| --- | --- | --- | --- | --- | --- | --- |
| Adanijo et al (2025) | UK | To explore YPs experiences of transitioning from CAMHS to AMHS and their views on improving the process | Qualitative interview study: semi-structured interviews | 8 had affective disorder, 5 of these had confirmed or suspected ASD | 8 YP: aged 18 to 20 years (mean age = 18.88 years, SD = 0.83 years). six were female and two were male. Four participants identified as white British, others as white Other (n = 2) or black British (n = 2) | Three main themes and nine subthemes. These included YP’s feelings concerning ‘leaving CAMHS behind’, their experience of ‘the transition process’, and ‘adjusting to adult services’ post-transition. Participants made six recommendations for improving transitions: providing more information about the process, greater involvement of YP and family members, more joint working between CAMHS and AMHS, better communication from AMHS, training clinicians to ensure AMHS foster age-appropriate and welcoming environments, and the development of an 18 to 25-year-old service. At least three of four optimal transition components were absent for 62.5% (n = 5) of the participants. |
| Allemang et al (2022) | Canada | To describe the transition readiness scores of YP with chronic health conditions with and without mental health comorbidity, and to examine the association between mental health comorbidity and transition readiness | A secondary analysis of data collected for the Transition Navigator Trial (TNT). All patients completed the Transition Readiness Assessment Questionnaire (TRAQ). | Youth with co‐occurring chronic health and mental health conditions (any) - same as Allemang et al 2023 | 201 participants: 140 (70%) participants had physical health conditions only (Cohort A), and 61 (30%) participants had co-occurring physical health and mental health conditions (Cohort B) - same as Allemang et al 2023 | The median TRAQ score for Cohort A was 3.87 (IQR 0.84) versus 4.00 (IQR 0.87) for Cohort B. Having a mental health comorbidity (b = 0.402, p = 0.034), being older in age (b = 0.540, p = 0.004) and being female (b = 0.388, p = 0.001) were associated with higher overall TRAQ. The presence of a mental health comorbidity was associated with greater transition readiness as measured by the TRAQ . |
| Allemang et al (2023) | Canada | To describe and explore the transition readiness of YP with co‐occurring chronic health and mental health conditions using TRAQ and qualitative methods | 1. Quantitative – cross-sectional (TRAQ: contains questions related to five subscales: managing medications, appointment keeping, tracking health issues, talking with providers and managing daily activities), 2. Qualitative interviews, 3. Integration | Co‐occurring chronic health and mental health conditions: rheumatological (36%), neurological (16%), endocrine (13%) and gastrointestinal/liver (13%); anxiety (82%), depression (57%) and (ADHD; 26%). | TRAQ sample: 61 participants; mean age 16.9 years, White (72%), Canadian-born (95%); Qual sample: 9 participants, mean age 18.3 years, White (67%), Canadian‐born (78%) | Several YP described developing transition readiness as a result of overcoming mental health challenges, engaging with a series of care providers (including mental health providers) and practicing self‐management skills in therapy. YP described using adaptive strategies including apps and symptom trackers to assist them in managing their medications. Results demonstrated the assessment of transition readiness using a generic scale does not address the nuanced and complex needs of youth with co‐occurring chronic health and mental health conditions. |
| Appleton et al (2021) | MILESTONE: UK | To explore the reasons why YP fall through the gap between CAMHS and AMHS, and what effect this has had on them and their families. | Qualitative interview study: narrative interviews; thematic analysis. | Neurodevelopmental or anxiety disorder, or depression | 15 YP: Mean age 19.42; 53% female; 90% White British; 5% British Asian; 5% Mixed; 15 parents | Participants represented 19 unique transition stories. Common reason for falling through the gap: not meeting the threshold for entry into AMHS; a group of YP not ill enough to access AMHS were too ill for other/non-specialist AWBS; barriers to access AWBS were the need for self-referral and the frequent use of telephone assessments to assess eligibility; reluctance of YP to see a new clinician and build a new therapeutic relationship; GP: some thought their GP did not have the appropriate expertise to manage their medication or make appropriate referrals; Falling through the gap had overwhelmingly negative effects on YP and their parents; problems with medication after leaving CAMHS. parents reported taking on a significant role in their child’s care. |
| Appleton et al (2022) | MILESTONE: UK | To explore the experiences and perspectives of YP and the parents/carers of YP receiving primary care support after CAMHS and to identify barriers and facilitators to accessing primary care. | Qualitative interview study: narrative interviews; thematic analysis. | Neurodevelopmental or anxiety disorder, or depression | 14 YP; 13 parents. Stories associated with 16 YP: Mean age 19.31; 56.3% female; 93.8% White British, 6.2% British Asian | Participants represented 16 unique transition stories. some YP. Some stated extremely positive experiences of accessing mental health support from their GP, whereas others, for various reasons, perceived their GPs as not supportive. Barriers included the perception that GPs couldn't prescribe certain medication, anxiety caused by the general practice environment, and having to move to a new practice at university. YP's positive experiences were more likely to include having a long-term relationship with their GP and finding that their GP made time to understand their needs and experiences. |
| Appleton et al (2023) | MILESTONE: Belgium, France, Germany, Ireland, Italy Netherlands, UK | To explore the predictors of YP falling through the gap versus transitioning to AMHS; to examine the healthcare and societal costs associated with transitioning and falling through the gap. | Longitudinal cohort study (MILESTONE) – secondary data analysis: T1 (baseline), T2 (+9 months), T3 (+15 months) and T4 (+24 months). | Anxiety or mood disorder, neurodevelopmental disorder or emerging personality disorder | 488 participants; Mean age at baseline 17.04; Female 58.2%, Male 41.6%; White European 80.94% | 336 were judged to have fallen through the gap, while 152 transitioned to AMHS. YP were more likely to transition to AMHS if they were severely ill. Those from Italy, the Netherlands and the UK were more likely to fall through the gap than transition to AMHS. Healthcare costs fell for all YP over the study, with a sharper decrease for those who fell through the gap. |
| Bisp et al (2022) | UK | To describe a service improvement project (Positive Pathways) which aimed to provide ‘step-down’ support for YP on discharge from a CAMHS (collaborative personalised care planning with a dedicated youth facilitator, social prescribing, and the provision of a personal health budget) | Quasi-experimental study: pre-post SWEMWBS survey | Any | 98 YP have been involved with Positive Pathways | 98 YP have been involved with the received and received a personal health budget, with 18 completed pre-post SWEMWBS. For all, post intervention score was higher than the pre-intervention score. |
| Boonstra et al (2024) | MILESTONE: Croatia, Ireland, Netherlands, UK | To learn from service users’ transition experiences and suggested improvements | Qualitative interview study: semi-structured interviews with MILESTONE study participants at second-year follow-up. | Any | 19 - age range: 18-24; 9 females | Main themes: abrupt changes in responsibilities, various barriers and a lack of preparation, communication and ongoing care. YP expressed a great need for continuity of care. Suggestions to improve transitional care included early and adequate preparation, joint working, improved communication from and between services, overlapping services, staying at CAMHS for longer and designated youth mental health teams. |
| Bravo et al (2020) | Spain | To describe the transition model from a sample recruited at CAMHS in Barcelona. | Cross-sectional study: descriptive analysis of a sample of adolescents referred from CAMHS to AMHS, regarding active follow up, waiting days and psychiatric characteristics | Any | 51 CAMHS patients: 58.8% males | Between January 2018 and June 2019, 51 CAMHS patients were referred to 3 different AMHS; 34 of them (66.6%) have an active follow-up. 23 patients (45.1%) meet criteria for severe mental disorder, and 34 (66.6%) have neuropsychiatric-treatment prescriptions. Sequential model was used in 45 (88.2%), whereas 6 patients (11.7%) needed parallel transition. Average waiting days until transfer was 49.9 days. One CAMHS-AMHS coordination-meeting takes place every 6 months to assure optimal transition. |
| Brikell et al (2024) | Australia, Denmark, Hong Kong, Iceland, the Netherlands, Norway, Sweden, UK, USA | To determine the rate of ADHD treatment discontinuation across the lifespan and to describe similarities and differences across countries to guide clinical practice | A retrospective, observational study using population-based registry data | ADHD | 1 229972 individuals; 735503 (60%) males, 494 469 females (40%); median age 8–21 years | Across countries, treatment discontinuation 1–5 years after initiation was lowest in children, and highest in young adults and adolescents. Within 1 year of initiation, 65% (95% CI 60–70) of children, 47% (43–51) of adolescents, 39% (36–42) of young adults, and 48% (44–52) of adults remained on treatment. The proportion of patients discontinuing was highest between age 18 and 19 years. |
| Canaway et al (2023) | MILESTONE: Belgium, Croatia, France, Germany, Italy, Netherlands, UK | To examine the use and cost of healthcare for YP engaged in mental healthcare before and after the child/adolescent and adult service boundary. | Pre-post descriptive economic analysis of  MILESTONE trial data at baseline (6 months before the service boundary) and 15 months later (post boundary); to capture resource use, a Client Service Receipt Inventory (CSRI) was adapted; the primary measure of mental health status was the HoNOSCA | Anxiety and/or depressive disorders (37%), behavioural disorders (35%), psychotic disorders (9%), feeding/eating disorders (7%), and trauma and stressor-related disorders (7%) | 772 YP attending CAMHS in seven European countries: 97 from Belgium, 52 from Croatia, 79 from France, 109 from Germany, 190 from Italy, 118 from The Netherlands and 127 from the UK | The proportion of YP engaging with healthcare services fell substantially after crossing the service boundary (associated costs €7761 pre-boundary v. €3376 post-boundary). Pre-boundary, the main cost driver was in-patient care (approximately 50%), whereas post-boundary costs were more evenly spread between services; cost reductions were correlated with pre-boundary in-patient care. Severity was associated with substantially higher costs pre- and post-boundary, and those who were engaged specifically with mental health services after the service boundary accrued the greatest healthcare costs post-service boundary. |
| Carles et al (2022) (French) | France | To understand the paediatricians’ and child psychiatrists’ experience of transition and to identify national practices around the transition process for adolescents and young adults with anorexia nervosa. | Qualitative interview study | Anorexia nervosa | Health care professionals: 13 paediatricians and child psychiatrists | Perspectives to improve the transition to adulthood care: early identification of the target population, choice of the most adapted unit to the severity level of the disease, family support during the transition, identification of a coordinating physician and enhanced networking support |
| Cleverley et al (2020) | Canada | To qualitatively explore transition-related knowledge and experiences of youth both prior-to and after transition. | Qualitative interview study | All youth reported having either a mood and/or anxiety disorder or which the majority were receiving treatment | 14 YP from LYiTS prospective cohort study. Another 8 youth were took part in post-transition interviews. | Youth advocated for being considered partners in transition planning and to have increased control over transition-related decisions. Youth also made specific recommendations on how to improve continuity of care during the transition process: Partner with youth in transition planning; Timing of transition is purposeful; Ensure access to transition related information; Ensure relational continuity between CAMHS and AMHS5; Additional programmatic recommendations; Recommendations for Other Youth and Their Families |
| Cleverley et al (2022) | Canada | To engage patients, caregivers, and clinicians to prioritize core components of successful CAMHS–AMHS transitions which can be used in the design or evaluation of transition interventions. | A modified e-Delphi method: initial survey items created based on an extensive literature review and input from expert advisors with lived experience; ranked by three expert panels based on importance and feasibility. | Youth participants: anxiety disorder (95.0%), with fewer reporting a mood (65%) and/or personality (30.0%) disorder | Round 1 Panel 1: 20 service users, specifically CAMHS users (aged 19–25) transitioned to an AMHS, 80% female, mean age 21.5; Panel 2: 17 caregivers of CAMHS users aged 19–25 who had transitioned to AMHS at the time of recruitment; and Panel 3: 21 clinicians from any professional designation (i.e. registered nurse, physician, etc.) and mental health administrators | A list of 26 core components of CAMHS–AMHS transitions has been refined which can be used in the design, implementation, or evaluation of interventions intended to improve transition experiences and outcomes for youth in mental health care. Core elements: Transition policy; Transition Tracking & Monitoring; Transition Readiness; Transition Planning; Transfer of Care; Transfer completion |
| Cleverley et al (2023) | Canada | The Transition Readiness Assessment Questionnaire (TRAQ) 5.0 is a 20-item questionnaire that measures transition readiness in youth preparing to transition to adult care. The objective of this study was to validate the TRAQ for use among youth accessing mental health services. | Cross-sectional psychometric validation of TRAQ 5.0, using the Longitudinal Youth in Transition Study (LYiTS) baseline cohort: internal consistency and convergent validity with the Dimensions of Emerging Adulthood (IDEAS) and Difficulty in Emotional Regulation (DERS) scales. | Anxious-depressed (48.1%), Withdrawn-depressed (33.3%), Attention Problems (28.7%), Thought Problems (24.4%), Social Problems (23.5%), Somatic Complaints (23.3%), Rule-Breaking Behaviour (10.8%) and Aggressive Behaviour (7.2%). | 237 clinically referred youth (aged 16–18 years) receiving outpatient mental health treatment; mean age of the sample was 16.8; 71.3% female | The CFA indicated adequate fit of the five-factor TRAQ structure. The overall scale (=.86) and three of the subscales demonstrated adequate internal consistency. As hypothesized, overall TRAQ scores were higher for youth who had discussed transition and those aged 18.The TRAQ 5.0 appears to be valid tool to assess the transition readiness of youth in outpatient mental health services. |
| Colizzi et al (2024) | Italy | To retrospectively identify the sociodemographic, anamnestic and clinical features of patients presenting to a transition-age youth mental health outpatient clinic ("PRIMA"), for individuals aged 16–35, during the first 24 months after its opening; (ii) to retrospectively profile their diagnostic-therapeutic process in terms of reasons for referrals and diagnostic and 6-month follow-up outcomes | Case note review through clinical records: including: 1. sociodemographic data; 2. Anamnestic data: (i) general medical history, (ii) past psychiatric history, (iii) family psychiatric history; 3. PRIMA clinical data: (i) reason for referral, (ii) referral route, (iii) diagnosis, (iv) therapeutic interventions, (v) outcome. | Any mental health condition; all consecutive patients who made at least one visit btw Nov 2021 and Oct 2023 | 99, 22.68 ± 5.201; ≤17 y-o: 10 (10.10%) (17, 20) y-o: 43 (43.43%) (20, 30) y-o: 36 (36.36%) >30 y-o: 10 (10.10%) | Many patients were referred by the general practitioner (25%) or were self-referrals (25%), while only a few patients came from child or adult mental health services (14%). Most had a comorbid physical condition (64%), with relatively less suffering from a neurological disorder (23%). Most patients presented with multiple reasons for referral (88%), mostly anxiety (56%), followed by affective symptoms (52%) and sleep problems (37%). Many patients presented with a request for diagnosis (28%). At follow-up, one in two patients were still under the care of the PRIMA outpatient clinic (52%). For a relevant proportion of patients, care was delivered by a specialist service. |
| Culnane et al (2022) | Australia | To describe carer perspectives of transition to adult care and the outcomes of a transition support intervention, Fearless, Tearless Transition, for adolescents with dual disabilities: a family-centred approach with early, purposeful planning. The shared care component: early engagement of GPs in shared care with paediatricians. | Pre-post survey evaluation of an intervention. | YP had a coexisting diagnosis of an ID and/or ASD with a mental health condition and were receiving care from a paediatrician, psychiatrist or subspecialist at the RCH. | Parents/carers attending clinic; 138 adolescents and their carers received support in a dedicated transition clinic with 99 carers completing the initial survey at the commencement of the model. Adolescents were eligible to receive the model of care if they were aged 12–18 years | 22 completed the post-evaluation survey: 67% of participants found the transition clinics to be at least somewhat useful or useful. Mean scores for preparedness for transition showed a modest but significant improvement at the postintervention follow up (4.6 ± 2.4 and 5.6 ± 2.6, P = 0.04), but there was no change detected in confidence to transition to adult care. "Given the high prevalence of psychiatric comorbidities in this patient group, it would be desirable to have a psychiatrist as part of the clinical team during transition preparedness." |
| Davies et al (2024) | Canada | To understand if there is an association between co-occurring physical health conditions and transition readiness and if this relationship is impacted by severity of mental health symptoms. | Cross-sectional secondary analysis of the Longitudinal Youth in Transition Study (LYiTS) baseline cohort | Any, were receiving services at either hospital-based (n = 137, 57.8%) or community-based (n = 100, 42.2%) organizations | 237 participants completed the baseline LYiTS surveys: aged 16 (n = 92, 38.8%), 17 (n = 110, 46.6%) and 18 (n = 35, 14.8%) years | In this sample of 16-to 18-year-olds receiving CAMHS, having a co-occurring PH condition was associated with increased transition readiness. There was no interaction between mental health symptom severity and co-occurring physical conditions |
| Davis et al (2023) | USA | To examine patterns of mental health service use during the transition to adulthood among autistic adolescents and young adults (AYA). | Cross-sectional study using clinical data pooled from 5 years, 2015–2019; six outcomes: (1) receipt of psychotherapy, (2) use of ED care associated with a psychiatric condition, (3) use of any psychotropic medications, (4) use of psychotropic polypharmacy, (5) experience of an ADE, and (6) use of both psychotropic medication and psychotherapy. | Autism spectrum disorder (ASD) | 529 autistic AYA. Just over half of the patients were ages 11–16. 17-21: 25.1% (133) | Patients had higher odds of having a psychiatric ED visit if they were ages 17–21 (AOR relative to ages 11–16: 2.28; 95% CI 1.20–4.34), had a complex chronic condition (AOR: 3.01; 95% CI 1.58–5.73), were receiving psychotropic medication(s) (AOR: 3.20, 95% CI: 1.28–7.98), and were receiving psychotherapy (AOR: 3.19; 95% CI: 1.80–5.65). Race and ethnicity were not significantly associated with ED visits or ADEs, but Black AYA were more likely to receive psychotherapy and less likely to receive psychotropic polypharmacy than Whites, and Hispanic AYA were less likely to receive any psychotropic medication(s) than non-Hispanics |
| Eke et al (2020) | UK | To explore views of clinicians working in both child and adult services for ADHD regarding the use of NICE guidelines for ADHD are discussed, with a particular focus on the processes and procedures that clinicians implement for transition between child and adult services. | Sequential mixed-methods study: a quantitative surveillance study informing qualitative telephone interviews | ADHD | Baseline survey: 202 paediatricians and 113 psychiatrists; Interviews: 22 clinicians from child services and 16 clinicians from adult services. Clinicians: mostly female (14:24 male to female ratio), either consultant paediatricians (n = 15), consultant psychiatrists (n = 19), or from other health disciplines (n = 4). | Information was shared between services in 85% of the 315 identified transition cases. A joint meeting was planned in 16% of cases; joint working before transfer occurred in 10% of cases. Clinicians were aware of NICE guidelines; they had mixed views on whether (local) guidelines or protocols were helpful. The main reason for not following guidelines was workload and resources: “NICE recommends stuff that is miles above what we will ever be able to provide”. |
| Fuchs et al (2016) | Austria | To demonstrate a) the frequency of admission of former child and adolescent psychiatry inpatients (CAP-IP) to adult inpatient mental health facilities, and b) a potential longitudinal diagnostic shift. | Retrospective longitudinal cohort study of inpatient cases at a specialized child and adolescent care centre with cross-sectional elements and registry linkage. Cases tracked using data matching with registry data from adult psychiatric institutions. Overall, observational period was 23 years. | 40 % of the 987 patients presented with psychiatric disorders with onset in childhood or adolescence, and about 1/3 presented with neurotic, stress-related and somatoform disorders. Approximately 5 % of presented with either psychoactive substance abuse, affective disorders or developmental disorders, respectively. | 1046 children and adolescents were admitted as inpatients at least once between 1 January 1989 and 30 June 2007. 987 patients were eligible for statistical analysis. Mean age at first admission was 13.8 years (SD 3.2 years), ranging from 2.83 to 17.98 years; male (48.5 %) and female (51.5 %) patients | More than a quarter of all former patients of a specialized facility for child and adolescent psychiatry were also inpatients at psychiatric clinics as adults. The likelihood in our sample of former CAP-IP also requiring psychiatric inpatient treatment in adulthood is 5.6 times higher than for the general Austrian population. First, older adolescents with several inpatient stays in short succession show a significantly higher risk of requiring inpatient treatment for a mental illness in adulthood. Second, 60 % of all CAP-IP with substance use problems show these same problems as inpatients in adulthood. Former child and adolescent psychiatry patients frequently develop addictive disorders, personality disorders, or adjustment and anxiety disorders in adulthood. |
| Gerritsen et al (2021) "Cohort profile…" | MILESTONE: Belgium, Croatia, France, Germany, Ireland, Italy, Netherlands, UK | To describe the demographic and clinical characteristics of YP in the MILESTONE cohort as they reach the upper age limit of their CAMHS. | Prospective longitudinal cohort study carried out in 39 CAMHS from 8 European countries. Participants were assessed via interviews and online questionnaires at baseline and follow-up at 9, 15, and 24 months | Any mental disorder or regular CAMHS user, with an intelligence quotient (IQ) over 70 or no indication of intellectual impairment | 763 YP from 8 European countries, 651 parents and 699 CAMHS clinicians. Age of recruited YP ranged from 15.2 to 19.6 years, with a mean of 17.5 years (SD=0.59) | The most common clinical classifications were depressive disorders (26.6%) followed by anxiety disorders (22.5%), attention deficit hyperactivity disorders (ADHD; 20.1%) and ASD; 14.9%.The mental health of YP reaching the upper age limit of their CAMHS varied greatly in type and severity: 32.8% of YP reported clinical levels of self-reported problems and 18.6% were rated to be ‘markedly ill’, ‘severely ill’ or ‘among the most extremely ill’ by their clinician. The duration of service use varied from less than 1year to more than 5years Fifty-seven per cent of YP reported psychotropic medication use in the previous half year. |
| Gerritsen et al (2022) "Demographic, clinical and service-use characteristics…" | MILESTONE: Belgium, Croatia, France, Germany, Ireland, Italy, Netherlands, UK | To examine which characteristics are associated with the clinicians’ advice to continue treatment at AMHS. | Prospective longitudinal cohort study, as above. | Any mental disorder or regular CAMHS user, with an intelligence quotient (IQ) over 70 or no indication of intellectual impairment | 763 YP from 8 European countries, 651 parents and 699 CAMHS clinicians. Age of recruited YP ranged from 15.2 to 19.6 years, with a mean of 17.5 years (SD=0.59), 60% female, 90.3% White | YP with higher clinician-rated severity of psychopathology scores, with self- and parent-reported need for ongoing treatment, with lower everyday functional skills and without self-reported psychotic experiences were more likely to be recommended to continue treatment. Among those who had been recommended to continue treatment, YP who used psychotropic medication, who had been in CAMHS for more than a year, and for whom appropriate AMHS were available were more likely to be recommended to continue treatment at AMHS. YP whose parents indicated a need for ongoing treatment were more likely to be recommended to stay in CAMHS. The recommendation to continue treatment at AMHS was mostly affected by service-use related characteristics, such as the availability of appropriate services. |
| Gerritsen et al (2022) "Leaving child and adolescent mental health services…" | MILESTONE: Belgium, Croatia, France, Germany, Ireland, Italy, Netherlands, UK | To discern the type of care YP receive after reaching the upper age limit of their CAMHS and examine differences in outcomes at 24-month follow-up between YP receiving different types of care | Prospective longitudinal cohort study, as above. | Any mental disorder or regular CAMHS user, with an intelligence quotient (IQ) over 70 or no indication of intellectual impairment | 763 YP from 8 European countries, 651 parents and 699 CAMHS clinicians. Age of recruited YP ranged from 15.2 to 19.6 years, with a mean of 17.5 years (SD=0.59), 60% female, 90.3% White | For YP, the higher their scores on the HoNOSCA (p=0·0009) and Youth Self- Report and Adult Self-Report (p=0·046), and who had a clinical classification of severe mental illness (p=0·0033), had suicidal thoughts or behaviours or self-harm (p=0·034), used psychotropic medication (p=0·0014), and had a self-reported or parent-reported need for continued treatment (p<0·0001) at baseline, were more likely to transition to AMHS or stay in CAMHS than to have care end. Overall, over 24-months, the mental health of YP improved, but 24·4% of YP reported an increase in problems calculated using the reliable change index, of whom 5·3% had a clinically relevant increase in problems. At 24-months, no differences in change in mental health problems since baseline were found between YP who used different types of care (CAMHS, AMHS, or no care). |
| Gerritsen et al (2024) | MILESTONE: Belgium, Croatia, France, Germany, Ireland, Italy, Netherlands, UK | To adapt and validate the On Your Own Feet – Transition Experiences Scale (OYOF-TES) in a sample of CAMHS users and describe YPs and parents’ experiences with transition and end of care at CAMHS. | Prospective longitudinal cohort study, as above. The OYOF-TES was adapted to a mental health setting and translated. An End Of Care (OYOF-EOC) version (self- and parent-report) was developed. | Any mental disorder or regular CAMHS user, with an intelligence quotient (IQ) over 70 or no indication of intellectual impairment | 763 YP from 8 European countries, 651 parents and 699 CAMHS clinicians. Age of recruited YP ranged from 15.2 to 19.6 years, with a mean of 17.5 years (SD=0.59), 60% female, 90.3% White | A total of 457 YP and 383 parents completed an OYOF-TES or OYOF-EOC. The Cronbach’s alphas of the OYOF-TES and OYOF-EOC parent/self-report ranged from 0.92 to 0.94. The two-factor structure was confirmed. The mean overall satisfaction reported by YP was 6.15 (0–10; SD=2.92) for transition and 7.14 (0–10; SD=2.37) for care ending. However, 26.7%-36.4% of YP were unsatisfied. The OYOF-TES and OYOF-EOC can be used reliably in mental healthcare settings to capture young people’s and parents’ transition experiences. The majority of YP and parents was satisfied with the process of transition and care ending, yet a third of YP had negative experiences |
| Gerritsen et al (2024) “The association between trajectories” | MILESTONE: Belgium, Croatia, France, Germany, Ireland, Italy, Netherlands, UK | To investigate whether self-reported trajectories of persistent psychotic experiences are associated with likelihood of transition to AMHS and mental health outcomes. | Prospective longitudinal cohort study, as above. Trajectories of self-reported PE (3 items) from baseline to 24-month follow-up were determined using growth mixture modelling (GMM). | Any mental disorder or regular CAMHS user, with an intelligence quotient (IQ) over 70 or no indication of intellectual impairment | 711 YP from 8 European countries with at least one assessment of PE at any timepoint, mean age of 17.5 years (SD=0.59), 60% female, 90.3% White | At baseline, 56.7% of YP reported PE. GMM identified 5 trajectories over 24 months: medium increasing (5.2%), medium stable (11.7%), medium decreasing (6.5%), high decreasing (4.2%), and low stable (72.4%). PE trajectories were not associated with continuity of specialist care or transition to AMHS. Overall, YP with PE reported more mental health problems at baseline. Persistence of PE or an increase was associated with poorer outcomes at follow-up. Conclusions. PE are common among CAMHS users when reaching the upper age limit of CAMHS. Persistence or an increase of PE was associated with poorer mental health outcomes, poorer prognosis, and impaired functioning, but were less discriminative for continuity of care. |
| Giacobini et al (2025) | Sweden | To evaluate care transition and medication use in young adults with ADHD in Sweden | Observational nationwide retrospective study of patients with ADHD from the Swedish National Patient Register, Prescribed Drug Register, and Cause of Death Register (2018–2020). Last contact with paediatric psychiatric care, first contact with adult psychiatric care, and medication use were described for ages 18 to 21 years, inclusive. | ADHD | 19,233 patients who had received dispensed medication for ADHD treatment. The majority of patients were male (57.1%), and 64.3% were aged 18 years at study entry. Patient’s birth month was evenly split across the year. The median follow up period was 2.5 (interquartile range [IQR] = 1.9–3.1) person-years | Of 19,233 patients who had dispensed ADHD treatment, 85.8% had received adult psychiatric care by 21 years of age. The proportion of patients on medication was highest at age 18 years (80.1%), decreasing to 36.1% by age 21 years. This study demonstrates declining ADHD medication use during the paediatric-to-adult care transition for patients in Sweden. |
| Gilsbach et al (2024) | Germany | To assess current and previous treatment for EDs during transition period and to determine how many patients in emerging adulthood had already managed the transition. To explore experiences and views of former inpatients during the transition process. | Qualitative interview study: Semi-structured interviews with former inpatients. | Anorexia nervosa | 32 YP who had been treated as inpatients during childhood and/or adolescence and come of age during the last 3 years; mean age 20.3 years; all female | Only approximately one‐third of the participants had already undergone the transition. Nearly 60% of the former patients were still cared for by a CAMHS, and only 12.5% had stopped treatment for AN. Approximately 60% were exclusively or additionally cared for by their general practitioner. More than 50% of the participants still lived with their parents. Approximately 90% of the participants who remained in a CAMHS expressed concerns about transitioning, mostly about losing their trusted therapist and the assumption of personal responsibility. |
| Girela-Serrano et al (2023) | Spain | To identify early clinical characteristics of adolescents with ADHD that predicts AMHS utilization in the years immediately after transition from CAMHS. | Prospective longitudinal cohort study: Analysis of data from YP with ADHD from a cohort of YP recruited at the age of 12–17 years, who, at the time of follow-up, were over 18 years old. | ADHD | 114 participants (64.4% males, 31.6% females) with a mean age of 14.44 (SD = 1.59) at the time of baseline data collection. At the time of follow-up, the mean age of the participants was 20.1 years; 84.2% Caucasian | Among AMHS users, hyperactivity/impulsivity measures were significantly more severe (t = 2.668, df = 112, p < .001), ADHD combined subtype diagnosis (χ2 = 4.66, df = 1, p = .031) was more frequent and dysregulation profile in the SDQ-P was also significantly higher (t = _2.497, df = 109, p = .014). Adolescents with ADHD are more likely continue their care under AMHS if they present more severe symptoms of hyperactivity/impulsivity and emotional dysregulation. |
| Gray et al (2024) | USA | To explore bringing transition readiness assessment into the mental health care of youth with co-occurring disorders. | Cross-sectional study using multi-informant assessment: 1) a questionnaire completed by the patient or parent, 2) a clinician administered formal assessment of the patient’s mental health transition readiness. Clinicians formally assessed youth mental health transition readiness using the TRXANSITION Index | Any | 101 transition-aged youth (M = 17.49 ± 2.73 years) ranging from age 12.37–24.24 years. mostly female (60.40 %, N = 61) and of Hispanic ethnicity (79.21 %, N = 80). Most of the sample (85.14 %, N = 86) received psychotherapy services, as opposed to medication-only treatment (14.85 %, N = 15). | Mental health transition readiness was highest in the following TRXANSITION Index domains: Ongoing Support (85.15 %), Adherence (78.38 %), and Trade/School (71.29 %). Regarding Ongoing Support, most patients identified themselves as the primary manager of their mental health care in adulthood and had a plan for their educational/vocational future (Trade/School). Although the overall score for Adherence was high, 66.35 % of youth reported sometimes missing a full day of medication (inclusive of non-psychotropic medications) and 46.15 % reported having trouble taking their medications daily. Mental health transition readiness was lowest in the following domains: New Health Care Providers (30.94 %), Rx/Medications (37.99 %), and Insurance (42.57 %). Clinicians reported that utilizing the transition readiness assessment helped them identify a knowledge gap in need of remediation for 95.10 % (N = 96) of their patients. |
| Hansen et al (2024) | Denmark | To examine which clinical and sociodemographic features predict transfer from child and adolescent mental health services to adult mental health services and if transfer is associated with prognosis. | Retrospective cohort study with registry-based outcome follow-up: outcome variables indicating one-year prognosis: (1) Days of hospitalization in AMHS, (2) number of contacts to psychiatric emergency ward, (3) suicide attempt, (4) suicide and (5) conviction | Various ICD-10 diagnoses; neurotic/attachment disorders (21.2%) and hyperkinetic disorders (18.9%). Only 4.9% of patients were diagnosed with schizophrenia and related disorders. | Population was identified as individuals with an open outpatient contact in CAMHS from the age of 16–17 years, who were discharged from CAMHS in the period from 1/1/06–10/05/15 aged 17-25. | Out of 27,170 Danish adolescents, 16% transferred to adult mental health services. Individuals were recorded as “transfer to AMHS” if they had an outpatient contact in AMHS from 6 months before to 12 months after discharge from CAMHS. Transfer was predicted by schizophrenia (OR 6.16; 95% CI 5.51–6.90) and personality disorders (OR 2.08; 95% CI 1.84–2.34), while hyperkinetic (OR 0.54; 95% CI 0.49–0.59) and pervasive developmental disorders (OR 0.42; 95% CI 0.31–0.58) decreased likelihood of transfer. Transfer was also substantially predicted by inpatient admission (OR 3.37; 95% CI 3.14–3.61) and psychiatric medication (OR 2.07; 95% CI 1.92–2.23). Transfer was associated with higher rates of inpatient admission to adult mental health services (IRR 5.83; 95% CI 4.37–7.77), more psychiatric emergency contacts (IRR 12.0; 95% CI 10.7–13.4), more convictions (IRR 1.40; 95% CI 1.23–1.59) and suicide attempts (IRR 5.70; 95% CI 4.72–6.90). Our results also support a tendency in existing research that especially patients with neurodevelopmental disorders do not receive care in AMHS. |
| Hendrickx et al (2020) | 36 European countries | To seek psychiatric trainees' opinions regarding training on transition and the knowledge and skills required for managing transition. | Cross-sectional survey: distributed to trainees residing in European countries | Any | 78 trainees: 81% were females, 26% were training to become a general psychiatrist, 32% an adult psychiatrist, and 40% a child and adolescent psychiatrist | 27% of trainees stated they have good to very good knowledge about the transition process. Theoretical training about transition was reported in only 17% of the countries, and practical training in 28% of the countries. Ninety-four percent of trainees indicated that further training about transition is necessary. |
| Hugunin et al (2023) | USA | To explore paediatrician and child/adolescent psychiatrists’ perspectives of the role of coordinated care for emerging adults with serious mental health conditions, particularly as they transition to adult care. | Qualitative interview study: Semi-structured interviews with online survey consisting of screening and demographic questions before the interview. | Emerging adults with serious mental health condition | 10 paediatricians & 11 child/adolescent psychiatrists. 17 White/Caucasian and 16 female. | Key barriers to continuous, coordinated care for youth with serious mental health conditions included poor communication systems between providers, no organized process for the transition from paediatric to adult care, state licensing laws (particularly impacting college-age youth), inadequate connection to community supports, and poor reimbursement rates for psychiatric care. Termination of primary care in young adults and inadequate medication side effect monitoring were described as key gaps in care. Mental health diagnoses often overlook the role of trauma and the impact of one’s underlying life experiences. To improve the lives of youth with SMHCs, the broader life environment must be considered, including complex family dynamics and socioeconomic adversity like poverty. Both paediatricians and psychiatrists emphasized the need for family driven care and parental support. Many providers described the necessity of youth being active in their care, especially beginning in adolescence and into young adulthood. |
| Janssens et al (2020) | UK | Our objectives were to explore how many YP with ADHD are in need of services as an adult, what adult ADHD services are available and how ADHD stakeholders experience transition from child to adult services. | Mixed methods study: 1) 12 month surveillance study; 2) mapping study to describe services; 3) qualitative interview study with patients, parents and clinicians. | ADHD | Interviews: 64 attention deficit hyperactivity disorder patients, 28 parents and 52 health clinicians | Among 315 individuals eligible for transition, 64% were accepted, but only 22% attended their first adult services appointment. An association between attention deficit hyperactivity disorder, education and continuance of medication into young adulthood, plus parent involvement and feeling prepared for transition and adult life with attention deficit hyperactivity disorder, influenced investment. However, even with investment, how accessible adult services are, how patient needs fit with the remit of the adult service and the level of patient information available affect transition outcomes. The results also highlight how general practitioners can end up as care co-ordinators during transition by default. |
| Kilicel et al (2022) | Switzerland | To (1) describe the architecture of CAMHS in Switzerland and (2) map the CAMHS/AMHS interface. | Cross-sectional service mapping survey: using the adapted European CAMHS Mapping Questionnaire (ECM-Q) assessing the architecture and functioning of CAMHS and the adapted Standardized Assessment Tool for Mental Health Transition (SATMeHT) to map CAMHS-AMHS interface | Any | A total of 26 child and adolescent services were approached; 6 replies were received. | Data were gathered from six cantons. Activity data and transition policies were comparable between Swiss regions and European countries. The transition occurred at the age of 18 years, civil majority, in each canton, and between 0 and 24% (3/7) and 25% and 49% (4/7) of YP were expected to transition. One canton (1/7) benefitted from written guidelines, at the CAMHS level only, regarding transition but none had guidelines for mapping CAMHS/AMHS interface even at the regional level. |
| Kunczicky et al (2023) | Austria | To assess the transition specific needs of 17 to 24-year-old patients. | Cross-sectional patient survey using the Transition Readiness and Appropriateness Measure (TRAM) | Any | 39 YP - 69,2% female; aged 17 -23 years, with a mean age of 18.8 years | 80% of the patients stated that they needed further care. ,Patient-related factors‘ and family support‘ were described as barriers to the willingness to transition. A significant connection was found between identity development and the willingness or need for transition (r= 0.431, p< 0.01), although this was more pronounced in relation to the need for transition (r= 0.821 p< 0.01). In addition, those affected stated that they were frequently and severely burdened by stress, 45% reported self-injurious behaviour, 48% suicidal thoughts or behaviour in the last six months. In terms of psychopathology, anxiety and depression were among the most common symptoms. In terms of functional impairment, the ,relationships‘ area was the most affected. |
| Leavey et al (2019) | UK, Northern Ireland | To examine the transition process to: (a) trace service user progression through service boundaries and (b) determine outcomes in terms of the referral process and level of engagement with services. Examine the extent that social class and aspects of family background were predictors of transition outcomes. | Retrospective cohort study with registry-based outcome follow-up: electronic records and case notes of YP eligible to transition to adult care over a 4-year period across five Health and Social Care NHS Trusts in Northern Ireland. | Any | 373 eligible for transition over the 48-month period- 225 females (60%) and 148 males (40%). | While a high proportion of eligible patients made the transition to adult services, very few received an optimal transition process, and many dropped out of services or subsequently disengaged. Clinical factors, rather than social class, appear to be more influential in the transition pathway. However, those not in employment, education or training (NEET) were more likely (OR 3.04: 95% CI 1.34, 6.91) to have been referred to Adult Mental Health Services (AMHS), as were those with a risk assessment or diagnosis (OR 4.89: 2.45, 9.80 and OR3.36: 1.78, 6.34), respectively. |
| Livanou et al (2021) "Transition outcomes…" | UK | To explore transition experiences and outcomes of YP discharged from adolescent medium secure services to adult and community settings. | Qualitative interview study: semi-structured interviews | Common MH problems | 13 YP aged 18–19 years, and 5 carers | Poor transition outcomes were for YP with the most severe mental health symptoms and those who committed serious offences. Three overarching themes were identified: (1) unsettling environmental factors within adult services; (2) experiences of transition management and preparation and (3) parental experiences of transition process and engagement with adult services. |
| Livanou et al (2021) "Challenges and facilitators…" | UK | To explore the views and experiences of key professionals involved in the transition process from six adolescent medium secure units to nine adult secure and community services in England. | Qualitative interview study: semi-structured interviews | Any | 34 key workers from 15 child and adolescent (N = 21) and adult (N = 13) forensic hospitals | Three primary themes were identified: (1) transition processes and preparation; (2) transition barriers and challenges; (3) success factors to transition. Key differences in adult and adolescent service care-models and lack of emotional and developmental readiness to moving onto adult-oriented settings constitute major barriers to positive transition outcomes. Practice and policy implications are considered to address the need for service transformations. |
| Livanou et al (2024) "Co-producing a complex psychosocial intervention…" | UK | To co-produce a complex psychosocial intervention: the moving forward intervention (MFi); to explore the potential challenges in the design and content of the proposed intervention. | Co-production of an intervention: all individuals involved in the co-production plan were treated as equal partners to mitigate power imbalances and potential biases towards the researchers | Any | 14 Advisory Groups with keyworkers (n = 17, 10 females), YP (n = 13, 5 females) and parents (n = 2, both female). Mean age for YP: 17 years - age range: 17- 19 years. | Three overarching co-produced themes pertinent to expectations in adult services, improving communication gaps between services and facilitating the letting go period emerged from the co-production phase. It was suggested the MFi is co-delivered by a peer with lived experience to build trust and create hopefulness among YP. The importance of promoting graded transitions through standardised procedures was highlighted. Related challenges are the loss of secure attachment relationships and the lack of involvement of parents and youth in decision-making during the transition process. Our findings showed that one of the hardest challenges that YP experienced were the loss of therapeutic relationships during the transition process. YP highlighted the fun time they had with keyworkers in adolescent secure services in contrast to the lack of empathy experienced from staff members in adult services. |
| Livanou et al (2025) | UK | To explore the lived experiences of YP with EDs, their carers, and mental health professionals (MHPs) during the transition from CAMHS to AMHS. | Qualitative interview study: semi-structured interviews conducted with YP with lived experience and carers | Eating disorders | YP (n=6) and carers (n=9), and two focus groups involving 12 mental health professionals; recruited across six NHS sites in England, including two adult and four adolescent specialist eating disorder services. | Four key themes: navigating the complexity of transitions, we need carers involved, the shadow of separation, and suggestions for the TEDYi intervention related to coping strategies and self-management. For YP with comorbid conditions the lack of tailored care compounded the difficulties of becoming independent. YP with conditions such as autism expressed feeling ill-equipped to navigate AMHS without sufficient support.  YP described the drop in carer involvement in AMHS as necessitating a sudden need to “take control” over their own. This sudden autonomy left others feeling overwhelmed, particularly in managing disordered thoughts. Carers noted the frustration stemming from confidentiality policies resulting in a lack of support and understanding for families, who are left in the dark regarding their loved one’s treatment. |
| Lockertsen et al (2020) “Experiences of patients…” | Norway | To explore patients’ experiences of the transition between CAMHS and AMHS. | Qualitative interview study: semi-structured interviews and one multi-step focus group - user-initiated. | Eating disorders | 10 participants with experiences with the transition between CAMHS and AMHS; all female with median age 22; range 19-29. | Four overall themes emerged regarding the transition process between CAMHS and AMHS: (1) “Being unprepared and alone in the transition process” describes how a lack of preparation for the transition between CAMHS and AMHS makes them feel alone and increases stress. (2) “It takes time to create a trusting relationship” describes how time influences patients’ trust in therapists and motivation for treatment. (3) “We are not all the same” describes how adolescents develop differently but are not treated differently despite their diverse ability to be self-sufficient. (4) “How they see me and treat me affects my hope for the future” describes the interaction between adolescents and therapists. |
| Lockertsen et al (2020) “Mental health professionals...” | Norway | To explore the experiences of professionals concerning the transition from CAMHS to AMHS. | Qualitative interview study: one multi-step focus group and two individual interviews with eight experienced health care professionals | Eating disorders | 8 mental health professionals with experience of treating patients with AN; 7 females. General practitioner (GP), school nurses, psychiatrists, psychologists, and psychiatric nurses were represented | Barriers experienced during the transition process were classified into four categories: (1) different treatment cultures that describe differences in how parents are included in CAMHS and AMHS; (2) mistrust between CAMHS and AMHS that can create a lack of collaboration and predictability for the patients’ transition; (3) Clinicians` factors such as lack of professional self-confidence can influence continuity of care for patients; and (4) lack of trust between services and not enough focus on building a new alliance in AMHS negatively influences the transition. |
| Lockertsen et al (2021) | Norway | To examine how parents experience the transition process from CAMHS to AMHS. | Qualitative interview study: semi-structured interviews | Eating disorders | 12 (3 fathers and 9 mothers) parents who had experienced the transition from CAMHS to AMHS - transitions of 10 YP were discussed. | Six categories represent the parents’ experiences of the transition: (1) the discharge when the child turns 18 years old is sudden; (2) the lack of continuity is often followed by deterioration and relapses in the patient; (3) the lack of involvement and information causes distress; (4) knowledge – an important factor for developing a trusting relationship between parents and clinicians; (5) parents have overwhelming multifaceted responsibilities; and (6) parents need professional support. |
| Malkov et al (2021) | UK | To evaluate what happens to patients with personality disorder during the transition process to help better understand their experience, and how it can be improved. | Retrospective clinical record review: clinical records from Tower Hamlet CAMHS, East London NHS Foundation Trust, were reviewed retrospectively from July 2018 to November 2019 | Personality Disorder | 41 cases that transitioned from CAMHS to AMHS were identified. | 36 were given a diagnosis by the CAMHS clinician at transition and 5 had no diagnosis assigned. No cases had a PD diagnosis made by the CAMHS clinician, however 1 case mentioned ‘PD traits’, 1 mentioned ‘EUPD’ as a possible differential and 2 cases were labelled as ‘emotional dysregulation’. 17 cases were found that met DSM5 criteria for PD diagnosis. Comparing transition experience of PD vs non-PD patients, the PD patients had a less optimal transition process. |
| Marchini et al (2023) | Belgium | To assess factors playing a role in transition-aged youth psychopathology and to define predictors. | A case-control observational study: YP aged 17 years old were recruited between June 2020 and December 2021, from both clinical population (CP) group and non-clinical settings | Any | 220 participants, aged 17 years old, female (69.1%), single (96.8%), and born in Belgium (82.3%). | Clinical data were all significantly different between CP and NCP groups. YSR scores were found statistically different according to group (p < 0.001), first-degree family history of psychopathology (p < 0.001), CTQ (p < 0.001), and FAD (p < 0.001). Predictive dimensional model suggested that TAY psychopathology can be predicted by group, CTQ and FAD. Significant positive correlation was found between YSR and HoNOSCA (rho = 0.81) and negative correlation between YSR and physical and psychological health (rho = −0.69 and −0.71, respectively). |
| Marchini et al (2025) | France | To examine the care trajectories of all patients who consecutively accessed an early intervention centre in Paris, over the course of 1 year; to investigate the presence of care discontinuities and assessing the role of the early intervention centre in facilitating the transition from CAMHS to AMHS. | Retrospective clinical record review | Any | n = 194; 57.2% males, with a median age of 20 years (17.5–22.5). The majority of patients were adults (≥18 years old) upon arrival at the C'JAAD (n = 131), with a median age of 21 years (19.5–22.5). Sixty-three patients (32.47%) were aged below 18 at the time of referral, with a median age of 16 years (15–17). | More than one-third of the sample had been hospitalised for psychiatric reasons (35.2%) prior to arriving at the C'JAAD. The median onset age of care in CAMHS was 14 (10.5–16) and the discontinuation age was 16 (14–17), with a median duration of 12 months (2.75–24). For adult patients who experienced a discontinuation of care, the median interval between the end of care in CAMHS and the beginning of the C'JAAD assessment was 36 months (28.5–57). |
| Martin et al (2020) | UK | To investigate the role of Digital Communication (DC), including smartphone apps, email and text, given the known barriers and facilitators of mental health service transitions to AMHS. | A secondary analysis of qualitative data collected for the Long-term conditions YP Networked Communication (LYNC) study. LYNC was designed as a mixed-methods case study undertaken in 20 NHS youth services | Any | Wider study: 165 YP aged 16–24 years, 13 parents, 173 clinical team members and 16 information governance specialists. LYNC data set two early intervention services were selected providing a total of 24 interview transcripts (5 YP and 19 staff). | DC were used successfully by YP and staff in ways that ameliorated known barriers to service transitions. They engendered responsibility in YP, promoted service access and contributed to client safety, particularly in times of crisis. DC risks included over-familiarity between YP and staff, and the possibility that messages could go unread. DC have the potential to facilitate trust and familiarity during and after transition to adult mental health services. |
| Markoulakis et al (2025) | Canada | To explore the perspectives of Transition-aged youth (TAY), family members, and system providers regarding the support that can be provided by navigation services during transitions in MHA care | Qualitative interview study: semi-structured 1-2-1 and FG interviews  with TAY, family, and providers | Any | 63 participants: (14 TAY, 26 family, and 23 service providers) Youth (71 % women) ranged in age from 17 to 29, with an average age of 23. Family members (88 % women) ranged in age from 48 to 61, with an average age of 54. Of both TAY and family participants, half (n=20) had accessed navigation services (e.g., through the FNP or other services). | Five themes emerged during data analysis: navigation to traverse difficult pathways, navigation to ensure appropriate and comprehensive care, navigation to sustain continuity of care, navigation to support informed care, and navigation to facilitate TAY and family involvement. "Navigation services are thus an important intermediary for service integration and can be a core component of an efficient, integrated system that effectively supports transitions in care (...) Navigation services in MHA care are focused on reducing barriers to care, providing client-centred support, and integration of care." |
| McClelland et al (2020) | UK | To estimate the proportion of patients in a CAEDS who use mental health services as young adults, (b) to delineate service utilisation following treatment in CAEDSs and (c) to identify factors in CAEDSs that predict service utilisation in young adulthood. | Retrospective clinical audit of a consecutive cohort attending a UK CAEDS over a 5-year period | Eating disorders | 322 patients (aged 13–17 years); the majority were White British (83%), 3% were of Asian origin and the remaining 14% had other ethnic backgrounds. | A total of 68.3% of CAEDS patients received no mental health treatment as young adults. Although 13% of people seen in a CAEDS had brief eating disorder treatment as young adults, 10% received longer/or more intensive eating disorder treatment. Overall, 10.8% transitioned directly to an AEDS and 7.6% were re-referred following discharge from CAEDS. In our sample, older age and increased use of CAEDSs predicted increased eating disorder treatment in young adulthood. |
| Mirzaian et al (2024) “Barriers to mental health care transition…” | USA | To explore barriers to transition from paediatric to adult health and mental health care and services for individuals with Intellectual and developmental disabilities (IDD) and co-occurring MH conditions, by eliciting the view of stakeholders, including disability advocates. | Cross-sectional survey: questionnaire with close- and open-ended questions. | Any | Participants were stakeholders associated with a Center for Excellence in Developmental Disabilities mailing list. 283 minus 6 not eligible due to being less than 18 years of age. Respondents to open-ended question: 105 | The social ecological model indicated a need for improvement in availability and delivery of services at the individual, family, provider, systems of care, and society levels to better support transition of paediatric to adult health and MH care for YYA with IDD and co-occurring MH conditions. Need for family training to prepare them for responsibilities related to transfer of care and involving parents with lived experience as “navigators". Need for jargon-free or plan language information, also in other languages spoken in the country. |
| Mirzaian et al (2024) “Health care transition recommendations…” | USA | To explore what types of assistance would be helpful for YP with IDD and co- occurring mental health conditions to transition to adult-focused health care | Same as above, focus on the open-ended question: “What do you think would be most helpful to assist YP with IDD and mental health conditions with the transition from child to adult health care (not including mental health care)?” | Any | 144 respondents - largest group of stakeholders were disabilities advocates (68; 47.2 %) followed by community-based service providers (45, 31.3 %) and health care providers (30, 20.8 %). | Individual level: Need for more individualized support, including peer support; need for advocacy and mentorship, need for resources around supportive decision making; Family level: Need for training and education about services, advocacy, financial needs, medical services, legal services, decision making, training for parents on how to assist their children into adulthood such as conservatorship and adult living; Provider level: Need for case management and navigation Need for professional training on how to better support individuals with IDD; Systems level: Need for coordination between agencies and systems Need for crisis intervention training Need for expanded availability of health care and MH care services for individuals with IDD. |
| Mooney et al (2023) | Canada | To engage patients about the value of developing educational supports for transition into adult medicine and determine how these supports should be structured to be most useful to young adults with eating disorders. | Qualitative interview study: semi-structured interviews | Eating disorders | Six young adults (5 females and 1 male) | All participants thought it would be helpful to have an educational resource. Three main themes and seven subthemes. Themes identified related to the unique challenges of transition for ED patients given the age of onset and cycle of symptoms; issues in adult care related to comorbidities and new level of autonomy; and the value of educational resources as both a connection tool and a benchmark. Participants also thought it would be useful to include in any educational resource a summary of their previous treatments, information regarding the transition process, a list of main healthcare providers they saw for their ED, a description of the differences and expectations of the adult system, a list of their follow up appointments, and a list of community and emergency mental health resources. |
| Mousatat et al (2025) | France | To describe and compare the care practices between CAP and AP services that serve the same geographic population in Paris; to detail the methods used to prepare for the transition between these two types of services. | Retrospective cohort study with consecutive inclusion comparing care settings: YP followed in CAMHS and AMHS. | Eating disorders DSM-5-TR consistent diagnosis of AN, atypical AN and/or BN | 182 adolescent patients (mean age: 18.1 years; 52.7% male) from the 17th district of Paris, | Diagnostic patterns showed a slightly higher prevalence of psychotic disorders in the AMHS group. Transition planning to AMHS was considered for only 11.3% of CAMHS patients; however, 27.7% of YP followed in AMHS had been referred by CAMHS. Age main criterion for considering a transition to adult services, with a clear majority (73.3%) of those transitioned being male and having had previous contact with CAMHS. Of those who transitioned, almost half (46.7%) had a diagnosis of developmental disorder, and over half were taking medication at the time of transition. A large proportion of YP (33%) preferred to remain within CAMHS. Clinical follow-up discontinuation with stable mental health accounted for 16.1% of cases, while discontinuation without stability accounted 19.3%, an area of concern. |
| Newlove-Delgado et al (2019) "YP with ADHD…" | UK | To understand GPs’ experiences of involvement with this group and explore their views on the roles and responsibilities of primary and secondary care in transition, to inform recommendations for policy and practice. | Qualitative interview study: semi-structured interviews with GPs across the UK | ADHD | 14 GPs | In the absence of a smooth transitions, many GPs became involved ‘by default’. GPs reacted by trying to identify suitable specialist services and were faced with the decision of whether to continue ADHD prescribing. Decisions influenced by perceptions that prescribing carried risks, and concerns over responsibility, particularly where specialist services were lacking. Variation in service availability, and tensions around how shared care works in practice. Implementation of NICE guidance is highly variable, with implications for GPs and patients. |
| Newlove-Delgado et al (2019) "Resumption of ADHD medication…” | UK | To examine the resumption of attention-deficit hyperactivity disorder (ADHD) prescriptions in early adulthood in YP whose ADHD prescriptions stopped in adolescence. | A retrospective cohort study: secondary analysis of data from the Clinical Practice Research Datalink (CPRD), a large UK clinical database run by the Medicines and Healthcare Products Regulatory Agency (MHRA) | ADHD - 395 (27.4%) had a prescription for a non-ADHD psychotropic medication, 348 (24.2%) had a recorded diagnosis of another psychiatric disorder, and 64 (4.4%) had a recorded learning disability. | 1440 YP- 1293 were male (89.8%), 395 (27.4%) had a prescription for a non-ADHD psychotropic medication at any point, 348 (24.2%) had a recorded diagnosis of another psychiatric disorder (excluding learning disability), and 64 (4.4%) had a recorded learning disability. | Of 1440 cases, 109 (7.6%) had their ADHD prescriptions resumed. Characteristics associated with an increased probability of resumption included female gender, learning disability, referral to adult mental health services, and prescription of antipsychotic medication. Only a small proportion of YP who stopped ADHD medication subsequently resumed their prescriptions in primary care. Those that did resume were a more complex group. |
| Nikula et al (2023) | Canada | To assess the feasibility of a new intervention designed to support YP and parents in the transition from paediatric eating disorder treatment to adult mental health services | Quasi-experimental single‐arm pre‐post study: quantitative survey, individual qualitative interviews and written reflections - content analysis | Eating disorder | At baseline, all consented adolescents (n = 10) were 17 years old (100%), and most were female (80%), White (80%), and had Anorexia Nervosa (80%). | On average, participants completed 75% of the expected components in under 3 months, with varied completion of each expected intervention component. Participants found the intervention convenient and helpful. Parents reported a significant decrease in guilt. No significant differences in YP self‐reported ED symptomology (assessed by EDE‐Q), transition readiness and self‐management skills (assessed by TRAQ and SMSAG‐Y). By 1‐month post‐transition, three adolescents transitioned to interim supports, and none started specialist adult treatment. Participants made multiple suggestions including providing a list featuring area‐specific providers (e.g., private care), a period of parallel care, earlier transition conversations, and resources for comorbid mental health concerns. |
| O'Hara et al (2020) | MILESTONE: Croatia, Ireland, UK | To examine the ethical values which people expect to underpin the transition between CAMHS and AMHS in Croatia, Ireland and the United Kingdom | Secondary qualitative analysis of focus groups (12) held to assist in the development of a psychosocial ethics questionnaire. FGs were held with members of local and national youth MH advocacy groups, and non-mental health local groups and national agencies working with YP | Any | 111 people aged 16–60 years of age (female N = 94, male N = 17). The majority of par-ticipants had experience of MH illness (31% with personal experience, 50% with a close family or friend) | Perpetuation of stigma, autonomy and decision-making were central themes as both enablers and deterrents of successful transition. The tension between professional (and at times parental) paternalism and young persons’ growing autonomy was well captured in the themes; (a) desired practice, (b) who should decide, (c) the process of decision-making and (d) potential harm(s). |
| O'Leary et al (2024) | Ireland | To explore experiences of young adults who have transitioned from CAMHS-AMHS, employing a phenomenological approach | Qualitative interview study: A cross-sectional, qualitative approach utilising semi-structured interviews and IPA | Any | 6 young adults; participants were aged between 18 and 21 years (M = 19.5 years; SD = 1.38); 4 female, 2 male; currently attending AMHS, having transitioned from CAMHS. | Findings indicate that the transition presents major challenges for young adults and can cause a deterioration in mental health, in the context of multiple concurrent life transitions, discontinuity of care, a dramatic culture shift in AMHS, and experiences of impersonal care. Transitioning from child to adult services entailed adapting to a service ethos of chronic mental illness, which did not align with these young adults’ recovery-oriented perspectives. Participants expressed their view that AMHS clinicians did not know how to approach their treatment due to a lack of available options or diagnosis-specific expertise, leading these individuals to question the validity of their suffering. |
| Pontoni et al (2022) | Italy | To explore factors associated with transition in a cohort of former inpatients of a Children and Adolescents Intensive Treatment Ward (CAITW) | Retrospective cohort study: socio-demographic and clinical features of patients previously admitted to CAITW were matched to AMHS data for those patients having reached age requirements. | Any - inpatient | 75.5% of 322 (n = 243) patients were born in Italy. Mean hospitalization age was about 15 years (15.1 years; DS 1.8). | 126 reached the age threshold for transfer to AMHS in the study period. The transfer rate was 50%. Two years after transition-age boundary, CAMHS-AMHS continuity of care was found in 40% and disengagement in 6% of cases. Longer and multiple hospitalizations, atypical antipsychotics prescription and a diagnosis of psychotic disorders were factors associated with short and intermediate RIT. A positive psychiatric family history was negatively associated with successful short and intermediate RIT. Diagnosis of psychosis and learning-supported school attendance were associated with long RIT. Young adults with a history of psychiatric inpatient admission as children or adolescents have a relatively high rate of transition to AMHS. |
| Price et al (2019) | UK | To describe the development of a seven step approach to map adult ADHD service provision in the UK | A service mapping study: 1. Defining the target service 2. Identifying key informants 3. Designing the survey 4. Data collection 5. Data analysis 6. Communicating findings 7. Hosting/updating the service map. | ADHD | Key informants included patients, carers, a range of health workers, and commissioners. | The seven step pragmatic method was effective for collating and communicating national service data about UK adult ADHD service provision. Using a broad definition of adult ADHD services resulted in an inclusive list of identified services, and allowed the definition to be narrowed to National Health Service (NHS) funded specialist ADHD services at data analysis, with confidence that few relevant services would be missed. |
| Pryjmachuk et al (2024) | UK | To develop a co-produced, evidence-based, general model of service design for CYP experiencing common mental health problems | Sequential, mixed-methods study: evidence syntheses (scoping and integrative reviews), a collective case study in England and Wales and interviews with stakeholders | Any | Six ‘young co-researchers’ – young adults with lived experience of mental health issues –were employed to work alongside the study’s substantive researchers. Study participants: 108 participants (41 CYP, 26 parents, 41 staff | The model-building phase identified rapid access, learning self-care skills, individualised support, clear information, compassionate and competent staff and aftercare planning as core characteristics of high-quality services. These characteristics were underpinned by four organisational qualities: values that respect confidentiality; engagement and involvement; collaborative relationships; and a learning culture. |
| Reaume et al (2021) | Canada | To examine associations between morbidity status and mental health care use among YP. | Cross-sectional secondary analysis of Canadian Community Health Survey-Mental Health (2012) for which Computer-assisted interviews were conducted in individuals' homes or by telephone | Any | 5,630 individuals aged 15-29 years | Individuals with comorbid physical health problems had higher odds of mental health care use for those with mental (odds ratio [OR] = 12.54 [7.07, 22.25]) and substance use problems (OR = 2.97 [1.75, 5.05]). While these estimates were higher than for individuals without physical comorbidity, differences were not statistically significant. For mental health care needs not being met, associations were found for individuals with mental (OR = 2.56 [1.24, 5.26]) or substance use problems only (OR = 2.48 [1.06, 5.82]). |
| Reed et al (2024) | UK | To explore the facilitators and barriers to the implementation of youth models of MH care across England (UK) | Qualitative interview study: Focus groups with 6 different services in England; services must have implemented an established model of youth mental health care | Any | 31 professionals (senior organisational managers,  commissioners, service managers, and clinicians) participated,  with a minimum of three participants per focus group | Seven key themes were identified: a clear rationale for doing things differently, for YP by YP, “building those relationships is key”, service identity development, resource and infrastructure, leadership at multiple levels, and valuing and developing staff. Effective communication and leadership, co-production and cross system collaboration contribute to successful implementation of youth models of MH care. |
| Reis et al (2021) | Belgium | To present socio-demographic and clinical data of a small sample of TAY with ASD facing the boundary between CAMHS and AMHS. To investigate innovative standardized tools to manage the transition process | Longitudinal cohort study: baseline sociodemographic data collection using self-rated questionnaires: HoNOSCA-SR, WHOQOLBREF, and Transition Readiness and Appropriateness Measure (TRAM) | ASD | 10 participants: age ranged from 17 to 18 years old. 70% male, born in Belgium (60%). 9 out of 10 were living with their parents, whereas only 1 with other people | The median (interquartile range) age of ASD diagnosis was 16 (13-17). The comorbidity rate was 60% for psychiatric disorders and 30% for somatic diseases; 80% of the participants were under psychotropic drugs. The average questionnaires scores were respectively: HoNOSCA-SR = 21.63 (±9.90), WHOQOL-BREF physical health = 50.88 (±19.14), psychological health = 36.13 (±13.97), social relationships = 48.50 (±18.23) and environment = 59.38 (±14.08); TRAM’s number of barriers to functioning were 4.25 (±1.39). |
| Reneses et al (2019) | Spain | To identify and characterize clinical pathways in patients in CAMHS at the transition age | Retrospective cohort study: individuals received treatment at CAMHS, data collection two years before transition and 6 months after treatment at AMHS | Any | No information - abstract only | Five clinical pathways were identified: 1: Referred to AMHS by CAMHS (29.2%): associated with previous hospitalizations, day hospital care, emergency room visits, pharmacological treatment and/or eating disorders. 3: Dropped out of CAMHS before transition (46,8%): associated with depressive, adaptive or anxiety disorders or to not having a diagnosis and/or no pharmacological treatment. 4: On-going treatment at CAMHS after transition age (9,7%): associated with psychosis, bipolar or serious neurodevelopmental disorders and/or previous hospitalizations or pharmacological treatment. |
| Reneses et al (2023) | Spain | To identify when and how patients get lost to care during transition by tracking care pathways and identifying factors which influence dropping out of care during transition. | A retrospective cohort study, same as above | Any | 760 patients who reached the transition age boundary within 12 months before transition time and were treated at CAMHS for at least 18 months. Median age was 17.83 years (interquartile range: Pc 75–Pc 50 = 18.2500–17.3333 = 0.9167). Female N= 402 | 46.8% of subjects (n = 356) withdrew from CAHMS without further contact with AHMS, 9.3% withdrew from CAHMS but were referred to AHMS by other services, 29% were transferred from CAHMS to AHMS, 10% remained at CAHMS and 5% patients were transferred to alternative services. Fifty-six percent of subjects experience cessation of care before the transition age. The risk of dropout increased with shorter contact time in CAMHS, was greater in subjects without pharmacological treatment, and decreased in subjects with psychosis, bipolar disorder, eating disorders, mental retardation, and neurodevelopmental disorders. |
| Rickwood et al (2023) | Australia | To examine changes in the key outcomes of psychological distress, psychosocial functioning, and quality of life for YP accessing headspace centres across Australis | Retrospective cohort study using routinely collected data: Data collection from YP commencing an episode of care (data collection period, 1 April 2019 to 30 March 2020), and at 90-day follow-up | Any | 58,233 YP aged 12–25 years first accessing one of 108 headspace centres for | Most presented with depression and anxiety issues (75.21%). Overall, 21.74% diagnosed with anxiety, 18.51% with depression, and 8.60% were sub-syndromal. Younger males were more likely to present for anger issues. CBT was the most common treatment. There were significant improvements in all outcome scores over time (P<0.001). From presentation to last service rating, over one-third had significant improvements in psychological distress and a similar proportion in psychosocial functioning; just under half improved in self-reported quality of life. Significant improvement on any of the three outcomes was shown for 70.96% . |
| Rickwood et al (2025) | Australia | To describe YP's session by-session experiences at headspace youth mental health services and examine how these varied over time and for different demographic groups. | Retrospective cohort study using routinely collected data: YP who commenced an episode of care at one of the 150 headspace centres between 1 July 2021 and 30 June 2022 and who had attended at least two services | Any | 16 484 YP aged 12–25 | Session ratings were more positive for young adults (18+ years) compared with adolescents (under 18 years). Scores on all five dimensions improved with more visits, and willingness to attend and working on issues important to the young person were strong predictors of service engagement. Better session experience scores were associated with more positive ratings of quality of life. |
| Roberti et al (2023) | Italy | To assess the transitioning process of ADHD patients in CAMHS and AMHS in the Italian territory; to report the current state of services and limitations and possible future matters that must be addressed. | A cross-sectional survey study: service-level surveys of number of ADHD patients, how many transitioning patients within the past year, and how transition is structured. Two surveys: CAMHS and AMHS. The two versions differed in questions about sending (CAMHS) or receiving (AMHS) patients | ADHD | 42 CAMHS, 35 AMHS | Out of 89 the CAMHS and 257 AMHS contacted, 77 centres (22% of those 346 contacted initially) filled in the survey (47% CAMHS and 14% AMHS) between December 2021 and May 2022. During the year preceding the survey, 36 CAMHS (85%) referred 15- to 21-year-old patients (Median = 18 years) to AMHS. Nonetheless, the desirable passage window would be narrower, ranging from a minimum of 17 years of age to a maximum of 19 years. The most frequent criteria that CAMHS used for patients’ selection for transitioning were age (83%) and clinical features (comorbidities, severe symptoms, and drug treatment) (31%).// On 7262 CAMHS patients, 206 transitioning patients were detected (around 3%). Indeed, this percentage is lower than reported elsewhere, at around 9% [14]. Still, appropriate information about the rate of transitioning ADHD patients, in particular in Italy, is scant. |
| Roberti et al (2024) | Italy | To enquire about the current transition paths in Italy and the perceived experiences of the patients and their clinicians | Mixed methods study: a) Qualitative semi-structured interviews with young adults with ADHD who turned 18 between 2017 and 2021. b) Cross-sectional survey of clinicians (both from paediatric and AMHS) | ADHD | Thirty-six interviews (18 from the north, 12 from the centre and 6 from the south) were conducted, in 13 cases with the participants’ parents (8 mothers and 5 fathers). The median age of the young adults at the time of the interviews was 22 years (SD = 1.3). | A referral to an AMHS was attempted for 16 young adults (8 before age 18 and 8 when turning 18), and 8 patients (22.2% overall) were successfully taken into the care of the AMHS. Twenty patients were not referred since it was deemed unnecessary (N = 6) or because of the lack of specialized services or compliance (N = 14). At the time of the interview, only nine participants were still under AMHS care. Other sentinel events were highlighted in the study, i.e., critical episodes that lead to emergency room access in the healthcare system, recurrent relational difficulties, reactivity and impulsivity, attentional difficulties, anxious symptomatology, as well as being unemployed. Unemployment also seemed common among those who were not referred to an AMHS but would have needed it. As already described, the risk of unemployment in ADHD young adults is high, even up to 70% (Helgesson et al., 2023). Episodes of fights, drunk driving episodes and car accidents must not go unnoticed. In our sample, these episodes were reported in 88% of the cases by these patients who were not in AMHS care. |
| Roberti et al (2024) | Italy | (1) To collect the available existing guidelines on the Italian territory and compare them with the eight points of the NICE guidelines, and (2) To identify the essential points that a practical and effective protocol in daily practice should address | Qualitative document analysis study: 21 services were asked to send a copy of their transition protocols to analyse their content via thematic analysis | ADHD | Six protocols were collected from the 21 centres that participated in the survey and reported a protocol, four from CAMHS and two from AMHS, none specific for ADHD patients. | In line with the NICE guidelines, the dimensions addressed in the 6 analysed documents are: early transition planning (although with variability in age criteria) (6/6), individualized planning (5/6), and the evaluation of transfer needs (5/6). All protocols also foresee joint meetings between child and adult services. The need to include the families is considered by 4 out of 6 protocols, while monitoring (2/6), and training programs (1/6) are less encompassed. In general, a highly heterogeneous picture emerges in terms of quality and quantity of regulations provided. |
| Rout et al (2023) | Germany | To investigate gaps in care during the transition to adult psychiatry | Standardised cross-sectional survey (phase 2 of sequential mixed methods study), in which patients were interviewed using a structured instrument focusing on help-seeking/service utilisation behaviour, the need for help and the experience during, before and after the transition phase. | Any | 100 AMHS patients with previous child and adolescent psychiatric treatment; 30 were inpatient; 70 outpatient; 57% female; 62% living with parents | A treatment gap of more than 3 months was documented in 75 % of the patients. The study showed that treatment interruption was experienced as a risk for further crises and that there was often a lack of information on further treatment options. Clear deficiencies were evident in the provision of care during the transition phase: a lack of professional support, and insufficient information for patients about follow-up treatment options in adult psychiatry. |
| Rowland et al (2026) | MILESTONE: Belgium, Croatia, France, Germany, Ireland, Italy, Netherlands, UK | To explore whether the use of managed transition (MT) can increase the proportion of YP making an appropriate transition from CAMHS to AMHS. | A two-arm cluster-randomised trial: Assessed at baseline, 9 months and 15 months, either by non-blinded research assistants or self-report measures (S. P. Singh et al., 2017) | Any | Total 793: Female 61.66%, Managed Transition: 241; UC: 552 | The use of MT lead to higher proportion of participants transitioning to AMHS compared to UC. This difference was not statistically significant across the whole trial. However, participants with a ‘transition requirement’ were twice as likely to transition to AMHS in the MT group compared to UC. There was no such increase in the group without a transition requirement. The intervention of managed transition led to a greater proportion of appropriate transitions to AMHS (i.e. in those with a pre-identified need for transition at baseline, or ongoing clinical need) without leading to an overall increase in inappropriate transitions in those without the need for AMHS. In the MT intervention arm, there were no transitions to AMHS among the group without a transition requirement. |
| Russet et al (2022) | 21 European countries | To describe the status of transition in adult psychiatry (AP) and child and adolescent psychiatry (CAP) postgraduate training in Europe; 2/ to explore the amount of cross-training between both specialties. | Cross-sectional survey study: 114-item questionnaire covering topics identified through a systematic review of the literature (23) and through the recommendations of the European Union of Medical Specialists (UEMS) and UEMS-CAP (31–35) | Any | 21 respondents, 50% were representatives of national psychiatry associations while 50% were from universities or research institutions engaged in training | Transition was a mandatory topic in the AP curriculum of 1/19 countries (5%) and in the CAP curriculum of 4/17 countries (24%). Most topics relevant for transition planning were addressed during AP training in 7/17 countries (41%) to 10/17 countries (59%), and during CAP training in 9/11 countries (82%) to 13/13 countries (100%). Depending on the training models, theoretical education in CAP was mandatory during AP training in 94% (15/16) to 100% of the countries (3/3); and in AP during CAP training in 81% (13/16) to 100% of the countries (3/3). Placements were mandatory in CAP during AP training in 67% (2/3) to 71% of the countries (12/17); and in AP during CAP training in 87% (13/15) to 100% of the countries (3/3). |
| Sadurni et al (2020) | Spain | To assess sociodemo-graphic and clinical characteristics of the CAMHS-AMHS population in the community mental health system in a neighbourhood of Barcelona. | A cross-sectional retrospective cohort study: descriptive analysis of a sample of patients who attended CAMHS and were referred to AMHS | Any | 28 patients; 64,3% male | 60,7% transitioned to AMHS; 39,3% were lost after finishing CAMHS. Parallel care was only applied to 1 patient. 46,4% of those transitioned to AMHS were diagnosed with a severe mental disorder; a third of them were lost to care during transition. The average time for a transition appointment in AMHS was 47,7 days |
| Salgado et al (2024) | Portugal | To characterize the perceptions of Portuguese physicians who are responsible for direct intervention in this process—child and adolescent psychiatrists (CAP) and adult psychiatrists (AP)—and to explore the possibilities for improvement. | Qualitative interview study: participants were selected using a convenience sampling of CAPs and APs in Lisbon area; thematic analysis. | Any | 28 APs and 18 CAPs expressed interest in participating. Saturation point was reached after interviewing eight CAPs and 13 APs. | Many YP transition informally to adult services. A formal and standardized transition process was not found. Two-thirds of the respondents thought that transition services were useful, while others thought that such services were not needed and had no positive effect on the quality of care. However, all agreed that in the absence of transition protocols, informal transfers provided the best results. -- All participants agreed on the importance of a seamless transition to ensure the continuity of care and that developing transition strategies to improve communication between paediatric and adult services would be beneficial. Some participants considered this issue for the first time. |
| Samuel et al (2025) | Canada | To compare the effectiveness of a patient navigator service to reduce emergency department (ED) use among YP and emerging adults with chronic health and/or mental health conditions undergoing transition to adult-oriented health care. | A pragmatic, parallel-group, nonblinded randomized clinical trial: Patients were followed up for a minimum 12 months and maximum 24 months after enrolment - At recruitment they were expected to be transferred to adult care within 12 months | Any + other chronic health condition/s | 335 participants were randomized over a period of 45 months, 164 (49.0%) to the intervention arm and 171 (51.0%) to usual care. After 1 patient withdrew, 334 participants (usual care: mean [SD] age, 17.8 [0.7] years; 99 female [57.9%]; intervention: mean [SD] age, 17.7 [0.6] years; 81 male [49.7%])were included in the final data analysis. | Among the participants, 131 (39.2%) resided in a rural location, and 126 (37.7%) had a self-reported mental health comorbidity during baseline assessment. We observed significant effect modification in the relationship between intervention and ED visits based on mental health comorbidity. Among those with a self-reported mental health condition, ED visit rates were lower in those with access to the navigator, but the association was not significant (adjusted incidence rate ratio [IRR] 0.75; 95%CI,0.47-1.19). Among those with no mental health comorbidity, the corresponding adjusted IRR was 1.45 (95%CI,0.95-2.20). The study did not accrue sufficient sample size to demonstrate a significant difference between groups should it exist. |
| Santosh et al (2020) | MILESTONE: Belgium, Croatia, France, Germany, Ireland, Italy, Netherlands, UK | To develop and validate the Transition Readiness and Appropriateness Measure (TRAM) for assessing a young person’s readiness for transition, and their outcomes at the transfer boundary. | A multi-phase instrument development and validation study, using mixed methods: 1) Literature review on transitioning; 2) review of important items regarding transition by a panel of 34 mental health experts. 3) Focus groups with YP; P/C; HCP. Two phase study: 4) The first phase involved 100 adolescents (pre-transition), young adults (post-transition), parents/carers and both CAMHS and AMHS clinicians. 5) The second phase involved over 1000 YP. | Any | Reported in supplement | A list of 64 items of potential importance were identified, which together comprised the TRAM. The psychometric properties of the different versions of the TRAM were evaluated and showed that the TRAM had good reliability for all versions and low-to-moderate correlations when compared with other established instruments and a well-defined factor structure.  This finding supports the use of TRAM to assess transition readiness, as higher correlations would imply that the TRAM was not adding anything new when compared with existing measures such as HoNOSCA. |
| Scanferla et al (2023) | France | To capture the subjective experience of eating disorder patients and their immediate family members in the transition between adolescent and adult treatment services and to explore how both groups make sense of this specific experience. | Qualitative interview study: in‐depth interviews using interpretative phenomenological analysis. Participants were recruited from a university department of a large psychiatric hospital specialising in eating disorders between July 2021 and September 2022. | Eating Disorders | A convenience sample of 18 participants was recruited, including 12 patients aged 19–30 years (m = 22.5, SD = 3.8) and six of their respective caregivers. | Four main themes were identified in relation to the participants' experience of transition to adult care: (1) the detailed description of the transition process, (2) the emotions associated with this experience, (3) the challenges encountered and (4) recommendations for improving the process. Two fundamental meaning‐making processes emerged: the feeling of being "lost in transition" and the opportunity to "become an adult". |
| Shahid et al (2025) | UK | To gain a better understanding of the barriers and facilitators discharging YP from community CYPMHS, by exploring clinicians’ experiences of discharging YP from their service. | Qualitative interview study: semi-structured interviews with clinicians working at different CYPMHS in England. | Any | 12, all females, primary mental health workers (33.3%) | Six themes were identified. These included, “perfectionistic approach towards treatment outcomes”, “reducing dependence on CYPMHS through empowerment”, “a lack of flexibility in the wider system”, “lack of collaborative care”, “an increasing pressure on the service” and “keeping the focus on discharge”. We found that discharges can be delayed at any age while in CYPMHS due to a number of factors. Clinicians, parents and voluntary sector organisations can hold a “perfectionistic” attitude, where they may delay discharge until they are satisfied that all of the young person’s mental health difficulties have been resolved. Families can also become very dependent on CYPMHS and be reluctant for their child to leave the service. Additional barriers to discharge included a lack of flexibility in the system and poor communication between all relevant parties. Lengthy waiting lists also made clinicians uncomfortable about discharging CYP, whilst high staff turnover caused further delays |
| Signorini et al (2020) | 16 European countries | To gather information on the characteristics and the involvement of social services supporting YP approaching transition. | Cross-sectional survey study: country respondents, representing social services’ point of view, completed an ad hoc questionnaire with 7 items. | Any | Respondents from 16/28 EU countries | Service availability ranges from a low of 3/100,000 social workers working with YP of transition age in Spain to a high 500/100,000 social workers in Poland, with heterogeneous involvement in youth health care. Community-based residential facilities and services for youth under custodial measures were the most commonly type of social service involved. In 80% of the surveyed countries, youth protection from abuse/neglect is overall regulated by national protocols or written agreements between mental health and social services, with the exception of Czech Republic and Greece, where poor or no protocols apply. Lack of connection between child and adult mental health services has been identified as the major obstacles to transition (93.8%), together with insufficient involvement of stakeholders throughout the process. |
| Singh et al (2023) | MILESTONE: Belgium, Croatia, France, Germany, Ireland, Italy, Netherlands, UK | To determine whether managed transition (MT) improves mental health outcomes of YP reaching CAMHS boundary compared with usual care (UC). MT included CAMHS training, systematic identification of YP approaching SB, a structured assessment (TRAM)and sharing of information between CAMHS and AMHS. | A two-arm cluster-randomised trial: clusters allocated 1:2 between MT and UC. Recruitment took place in 40 CAMHS in 8 European countries | Any receiving treatment or had a diagnosed mental disorder, had an IQ ⩾ 70 and were within 1 year of reaching the SB. | 793 were available for the baseline assessments; 61.66% Female; 83.23% White | The mean difference in HoNOSCA scores between the MT and UC arms at 15 months was −1.11 points (95% confidence interval −2.07 to −0.14, p = 0.03). The cost of delivering the intervention was relatively modest (€17–€65 per service user). MT led to improved mental health of YP after the service boundary but the magnitude of the effect was small. The intervention can be implemented at low cost and form part of planned and purposeful transitional care. |
| Street et al (2023) | MILESTONE: Croatia, Ireland, Netherlands, UK | To explore how YP in CAMHS in the UK, Ireland, The Netherlands and Croatia, experience leaving CAMHS and identify a range of factors impeding optimal discharge or transition to AMHS. | Qualitative interview study: Semi-structured interviews about discharge or transition planning, including what information was provided about their ongoing mental health needs. | Any | 34 YP aged 17–24, all previous or current attendees of CAMHS. Some interviews included accounts by parents or carers | A number of previously well-documented barriers to a well-delivered discharge or transition were noted. Two issues less frequently reported on were identified and further discussed; they are the provision of an adequately explained, timely and appropriately used diagnosis and post-CAMHS medication management. Overall, planning processes for discharging or transitioning YP from CAMHS are often sub-optimal. Practice with regard to how and when YP are given a diagnosis and arrangements for the continuation of prescribed medication appear to be areas requiring improvement. |
| Toulany et al (2019) | Canada | To analyse the association between primary care continuity during the transition from paediatric to adult care and need for acute mental health services in young adulthood | Longitudinal retrospective cohort study using linked health and demographic administrative data: all YP aged 12 to 16 years with severe mental illness between April 1, 2002, and April 1, 2014, in Ontario, Canada, followed up through March 31, 2017. | Severe mental illness i.e.. hospitalization for schizophrenia, eating disorder, or mood disorder | 8409 YP with severe mental illness (5720 [68.0%] female; mean [SD] age, 14.8 [1.2] years), 5478 (65.1%) | 5478 (65.1%) YP had continuous primary care, 2391 (28.4%) had discontinuous primary care, and 540 (6.4%) had no primary care during the transition period. YP with no primary care during transition were more likely to be male (57.2%), have lower socioeconomic status (31.5%), and have no usual primary care practitioner at baseline (25.6%). Compared with continuous care, patients with discontinuous and no primary care had an increased rate of mental health–related hospitalization in young adulthood (adjusted relative rate, 1.20; 95%CI, 1.10-1.30; and adjusted relative rate, 1.30; 95%CI, 1.08-1.56, respectively). Fragmented or no primary care during transition is associated with increased mental health admissions and emergency department visits after transition to adult care services. |
| Tunesi et al (2019) | Italy | To provide an estimate of the prevalence rate of ASD in 8-year-olds in 2017 based on administrative databases and to investigate the change in healthcare service use during the healthcare transition age of 18. | A longitudinal retrospective cohort study: data drawn from the Italian Administrative Healthcare Database (2010–2017). | ASD - Prevalence of ASD at age 8 was 5.4/1000 | From 5607 ASD patients, 331 ASD patients from 2012 to 2015 in the calendar year of their 18th birthday were selected and their health service utilisation during a 5-year period—ranging from 2 years preceding and succeeding their 18th year | Global access to health and social services was lower both before and after age 18 (46.5% at 16; 68.0% at 18; 54.1% at 20). The percentage of patients receiving a neuropsychiatric consultation decreased after age 18 (30.8% at 18; 5.4% at 20). Community mental health services (CMHS) utilisation rate increased above 18 years of age. Regarding psychiatric visits, for both outpatient and CMHS, an increase was observed from 17.8% at age 18 to 25.4% at age 20. The utilisation of rehabilitation services decreased with age, dropping from 17.8% at age 16 to 1.8% at age 20. Psychiatric outpatient services remained stable across ages at about 14%. |
| Vakili et al (2024) | Canada | To investigate consensus preferences among YP on important content, technology features, and engagement supports to inform a transition-focused SMS text message service | A modified e-Delphi survey: for youth in Canada aged 16-26 years who have accessed mental health services within the past 5 years. | Any | A total of 100 participants, predominantly non-White (n=47, 47%), aged 20-26 years (n=59, 59%), and who had first accessed mental health services between the ages of 13 and 19 years (n=60, 60%), were selected. | The content item with the highest level of consensus (88/100, 88%) was “the description of what to expect in your first adult mental health service appointment.” Other information-focused items that met the high consensus threshold were “contact information for who youth can speak to” (79/100, 79%), “a list of potential benefits for completing steps in the transition process” (74/100, 74%), and “a description of the role of health care providers in the transition process” (73/100, 73%). All 3 of the behaviour-focused items met the high level of consensus threshold: “link to the location or address of the adult community mental health clinic youth will be visiting” (81/100, 81%), “reminders or notifications about upcoming dates or actions” (78/100, 78%), and “demonstration of how to prepare for the first adult appointment” (76/100, 76%). |
| van Bodegom et al (2023) | MILESTONE: Belgium, Croatia, France, Germany, Ireland, Italy, Netherlands, UK | To investigate whether risk factors for persistent depression are considered in clinicians’ advice about continued treatment for depressed YP facing the upper age limit of their CAMHS. | Prospective longitudinal cohort study: CAMHS users from 39 CAMHS in Europe. At baseline, clinicians indicated on the Transition Readiness and Appropriate Measure (TRAM; [29]) what type of care they considered most appropriate for the young person. | Depressive disorder | First sample: N = 203; 72.9% female and 77.8% White: YP in CAMHS who were classified with unipolar depressive disorder by their clinician. Second sample: N = 185; 76.8% female and 77.2% White: YP with self-reported clinical levels of depressive problems over the past six months. | Only clinician rated severity of psychopathology was related to a recommendation to continue treatment for those classified with unipolar depressive disorder (N = 203; OR = 1 45, 95% CI (1.03–2.03), p = 044) and for those with self-reported depressive problems on the Achenbach DSM-oriented Depressive Problems scale (N = 185; OR = 1 62, 95% CI (1.12–2.34), p = 012). Contrary to the NICE transition guidelines, self-reported need for continued treatment does not seem to influence this recommendation. Furthermore, none of the risk factors for persistent depression and factors associated with a transition recommendation in general were related to a recommendation to continue treatment in AMHS rather than in CAMHS. |
| van Bodegom et al (2023) | MILESTONE: Belgium, Croatia, France, Germany, Ireland, Italy, Netherlands, UK | To study clinicians' and parents' awareness of suicidal behaviour in adolescents reaching the upper age limit of their CAMHS and its association with mental health indicators, transition recommendations and mental health service use. | Prospective longitudinal cohort study: CAMHS users from eight European countries were assessed using multi-informant and standardised assessment tools at baseline and nine months follow-up. | Any | 763: 60% female, mean age 17.50 (sd 0.59), 75.8% white; severe mental disorder 13.1%, behavioural/ neurodevelopmental disorder 59.6%, emotional disorder 59.6% | 53.5 % of clinicians and 56.9 % of parents were unaware of YP's self-reported suicidal behaviour at baseline. Compared to those whose clinicians/parents were aware, unawareness was associated with a 72–80 % lower proportion of being recommended to continue treatment. Self-reported mental health problems at baseline were comparable for YP whose clinicians and parents were aware and unaware of suicidal behaviour. Clinicians' and parents' unawareness were not associated with MHS use at follow-up. |
| van Bodegom et al (2025) | MILESTONE: Belgium, Croatia, France, Germany, Ireland, Italy, Netherlands, UK | To identify trajectories of self-reported depressive problems (ASEBA Youth Self-Report and Adult Self-Report) over a 24-month follow-up period, as well as associated risk factors for persistent depression and service use, using growth mixture modelling | Prospective longitudinal cohort study, as above | Depressive symptoms | At baseline, nine, 15- and 24-months follow-up, 89.5 % (n = 683), 73.1 % (n = 558), 66.7 % (n = 509) and 62.6 % (n = 478) of young people respectively provided information on self-reported depressive problems. | We identified a low, a decreasing, an increasing and a high trajectory of depressive symptoms. The high trajectory could be predicted with almost all risk factors for persistent depression (i.e. higher clinician-rated psychopathology, more suicidality, comorbidity and being a victim of bullying) and these YP were likely to transition to AMHS or require specialist mental health care later. YP whose depressive symptoms increased to similar levels as the group with persistent high levels of depressive symptoms (the increasing trajectory) could not be properly distinguished from other YP based on risk factors, while their needed continuation of treatment (either in AMHS or CAMHS) was equally likely as the end of care. |
| Winston et al (2023) | UK | To establish how many CAMHS teams meet recommendations for managing transitions between child and adolescent mental health services (CAMHS) and adult services for eating disorders  treatment in England. | Cross-sectional survey targeting services: A self-report questionnaire was designed for the study by the research team, based on the RCPsych’s guidance | Eating disorders | 38 ED services | Of the 38 services that participated, 31 (81.6%) reported a flexible upper age limit for treatment. Only 6 services (15.8%) always transferred YP to a specialist adult eating disorders service and the majority transferred patients to either a specialist service or a community mental health team. Most services complied with recommended provision such as a written transition protocol (52.6%), individualised transition plans (78.9%), joint care with adult services (89.5%) and transition support for the family (73.7%). |
| Wittevrongel et al (2023) | Belgium | To explore existing (dis)agreement regarding youth-friendly MH services and feasibility for implementation in clinical practice. Young adults (18–26 years) and parents were part of a public panel and professionals were allocated to the professional panel | A mixed‐method Delphi study: three online questionnaire rounds and a physical meeting. | Any | Most young adults participating in the study were female (89%), mean age 21.3 years (SD = 2.3) | Only nine out of the 47 consensus items (19%) were deemed to be highly feasible for clinical practice (a score of 7 or above on a 9‐point Likert scale as endorsed by ≥70% of the panellists) by both panels. This included the provision of an information brochure (1.3), a referral being unnecessary unless the service is highly specialized (1.6) and inviting the young person before the start of treatment (1.9). Offering free WIFI (1.13), creating a pleasant entrance/waiting area (1.14), having a cozy communal area (1.16) and a quiet room (1.26) were not only considered highly relevant to the youth‐friendliness of a service but also highly feasible. Furthermore, both panels thought working with a care plan (1.31) and organizing a network meeting before the end of treatment (1.41) were easy to implement. |

Abbreviations: YP – Young people; CAMHS - Child and Adolescent Mental Health Services; AMHS - Adult Mental Health Services; UK – United Kingdom; ASD – Autism Spectrum Disorder; ADHD – Attention Deficit Hyperactivity Disorder
